# Candidate Gene Polymorphisms Influence the Susceptibility to Salt Sensitivity of Blood Pressure in a Han Chinese Population: Risk Factors as Mediators

**DOI:** 10.3389/fgene.2021.675230

**Published:** 2021-10-04

**Authors:** Yunyi Xie, Zheng Liu, Kuo Liu, Han Qi, Wenjuan Peng, Han Cao, Xiaohui Liu, Bingxiao Li, Fuyuan Wen, Fengxu Zhang, Ling Zhang

**Affiliations:** Department of Epidemiology and Health Statistics, School of Public Health, Capital Medical University, Beijing Municipal Key Laboratory of Clinical Epidemiology, Beijing, China

**Keywords:** salt sensitivity, acute salt loading, blood pressure, single-nucleotide polymorphism, mediation analysis

## Abstract

Genome-wide association studies suggest that there is a significant genetic susceptibility to salt sensitivity of blood pressure (SSBP), but it still needs to be verified in varied and large sample populations. We attempted to verify the associations between single-nucleotide polymorphisms (SNPs) in candidate genes and SSBP and to estimate their interaction with potential risk factors. A total of 29 candidate SNPs were genotyped in the 2,057 northern Han Chinese population from the Systems Epidemiology Study on Salt Sensitivity. A modified Sullivan’s acute oral saline load and diuresis shrinkage test (MSAOSL-DST) was used to identify SSBP. A generalized linear model was conducted to analyze the association between SNPs and SSBP, and Bonferroni correction was used for multiple testing. Mediation analysis was utilized to explore the mediation effect of risk factors. Eleven SNPs in eight genes (PRKG1, CYBA, BCAT1, SLC8A1, AGTR1, SELE, CYP4A11, and VSNL1) were identified to be significantly associated with one or more SSBP phenotypes (*P* < 0.05). Four SNPs (PRKG1/rs1904694 and rs7897633, CYP4A11/rs1126742, and CYBA/rs4673) were still significantly associated after Bonferroni correction (*P* < 0.0007) adjusted for age, sex, fasting blood glucose, total cholesterol, salt-eating habit, physical activity, and hypertension. Stratified analysis showed that CYBA/rs4673 was significantly associated with SSBP in hypertensive subjects (*P* < 0.0015) and CYP4A11/rs1126742 was significantly associated with SSBP in normotensive subjects (*P* < 0.0015). Subjects carrying both CYBA/rs4673-AA and AGTR1/rs2638360-GG alleles have a higher genetic predisposition to salt sensitivity due to the potential gene co-expression interaction. Expression quantitative trait loci analysis (eQTL) suggested that the above positive four SNPs showed cis-eQTL effects on the gene expression levels. Mediation analysis suggested that several risk factors were mediators of the relation between SNP and SSBP. This study suggests that the genetic variants in eight genes might contribute to the susceptibility to SSBP, and other risk factors may be the mediators.

## Introduction

Salt sensitivity of blood pressure (SSBP) is defined as a quantitative trait in which the BP of some members of the population displays changes that are parallel to changes in sodium ingestion (Elijovich et al., 2016). The Genetic Epidemiology Network of Salt Sensitivity (GenSalt) study reported that the prevalence of salt sensitivity (SS), which is defined as a qualitative trait of SSBP, is approximately 34.2% among Chinese adults (Liu et al., 2020). SSBP not only plays a role in the pathogenesis of hypertension but is also a risk factor for cardiovascular complications that increase mortality. A 30-year follow-up study indicated that SSBP was a risk factor of mortality for both normotensive and hypertensive subjects (Weinberger, 2002). Approximately 33% of the variance in SSBP has been estimated to be mediated by genetic components (Gu et al., 2007).

Genes can be involved in SSBP pathology through various pathways (the renin-angiotensin-aldosterone system, ion and water channels, transporters and exchangers, the endothelial system, the apelin–APJ system, the sympathetic nervous system, intracellular messengers, and the kallikrein-kinin system) (Liu et al., 2020). Several genome-wide association studies (GWAS) and candidate gene studies have been conducted for SSBP, and the results were reviewed (Liu et al., 2020). Two GWAS have been conducted on SSBP subjects identified by acute salt loading and a chronic sodium diet (Citterio et al., 2011; Li et al., 2016). The GWAS conducted by Lorena Citterio on Caucasians with mild hypertension found that SNPs located in the cGMP-dependent protein kinase 1 (PRKG1) gene are associated with changes in the diastolic BP (DBP), whereas SLC24A3 and SLC8A1 are associated with changes in the systolic BP (SBP), which focuses on acute salt loading but that study is limited by modest sample sizes (*n* = 329), and the results may not apply to other racial groups (Citterio et al., 2011). However, the GenSalt study focuses on genetic determinants that may affect BP changes during chronic sodium loading in Chinese individuals using a family based, dietary-feeding study (Li et al., 2016). Although the GenSalt study did not identify any associations with some traditional candidate genes, it provided evidence for the involvement of novel genes such as UST, CLGN, AC079598.3, and RPS4XP15 (Li et al., 2016). Several candidate studies have also indicated that genomic variations are associated with SSBP; however, these results are inconsistent and limited considering that there are only a handful of SNPs in one gene or are limited by their statistical power. Such an association study is unable to explain the biological complexity of SSBP, a multifactorial disease with several components involved in its pathological mechanism, including numerous environmental, and genetic risk factors.

Therefore, replication studies of candidate genes from independent samples should be conducted to further validate these results. In our previous studies (Liu et al., 2017, 2018), we validated the genetic variations in several candidate genes, but these variations were limited to subjects with hypertension in a relatively small sample size (*n* = 342). Ultimately, the detection of variants in specific genes that participate in BP changes during acute salt loading and diuresis shrinkage in a larger Chinese sample will be needed. Moreover, SNPs and SSBP have a non-linear relationship, and potential risk factors may modulate the effects of SNPs on SSBP. The evidence has shown that several risk factors, including obesity, hyperglycemia, and dyslipidemia, are strongly associated with SSBP (Chen et al., 2009; Liu et al., 2020). Of note, several genes have been identified that are related to the above risk factors, which overlap with the genes associated with SSBP (Yazdanpanah et al., 2007; Ochoa et al., 2008; Niemiec et al., 2011; Ding et al., 2018). However, whether individuals with different genetic predispositions influence SSBP through the above intermediate phenotypes still lacks research evidence.

In the present study, we aimed to identify the associations between SNPs in candidate genes and SSBP, and their interactions with risk factors in SSBP susceptibility. We selected the pathogenic pathway of 29 variants in 23 candidate genes. in SSBP by predictions of their functional roles and previous genetic studies. A modified Sullivan’s acute oral saline load and diuresis shrinkage test (MSAOSL-DST) was utilized to distinguish SSBP. Genetic susceptibility to the changes in BP responses to the two intervention stages was analyzed. The relationships between SNPs and the potential risk factors were investigated, and we utilized a mediation analysis to detect the risk factors for the mediation effect on the associations between SNPs and SSBP.

## Materials and Methods

### Study Subjects and Sample Collection

A total of 2,057 unrelated participants were selected from the Systems Epidemiology Study on Salt Sensitivity (EpiSS). This study enrolled adults between July 2014 and July 2016 to identify the environmental and genetic risk factors for SSBP in northern China, and the protocol has been previously published (Qi et al., 2018). In brief, participants who met the following criteria were recruited in this study: (1) 35–70 years old; (2) unrelated Chinese Han people who were permanent residents of Beijing or Liaoning Province for more than 5 years in 13 communities; and (3) if the participant had hypertension, only patients diagnosed with early stage of essential hypertension [160 mmHg > SBP ≥ 140 mmHg and (or) 100 mmHg > DBP ≥ 90 mm Hg, according to the definition standard of the 2010 Chinese hypertension guidelines (Liu and Writing Group of Chinese Guidelines for the Management of Hypertension, 2010)] were included. Participants were excluded from this study if they were diagnosed with a stage two or more severe essential hypertension to avoid the risk of increased blood pressure during the acute salt loading; pregnant; or diagnosed with severe coronary disease, stroke, myocardial infarction, kidney disease, liver disease, or a tumor. MSAOSL-DST was used to evaluate SSBP (Liu et al., 2017). All research subjects used a unified questionnaire to investigate their salt sensitivity, including their general conditions and lifestyles. We investigated the self-reported salt-eating habit of the participant by asking the question “How salty do you usually eat your food?,” subjects could select from these answers: “eat salty,” “on average, eat salty,” and “eat sparingly.” We also collected the self-reported average daily walking time of the participants in the past month. DNA samples were available for 2,057 participants, and their demographic characteristics, lifestyle factors, medical case history, medical family history, and body measurement information were collected. This study was approved by the Ethical Committee of Capital Medical University. All participants signed an informed consent before participating in this study.

### Assessment of Salt Sensitivity of Blood Pressure

The evaluation of SSBP was conducted by MSAOSL-DST, the details of which have been previously published (Mu et al., 1993; Liu et al., 2017, 2018; Zhang et al., 2020). Each fasting participant received an oral administration of 1,000 ml of 0.9% saline solution within 30 min and oral administration of 40 mg furosemide after 2 h of saline loading. After a 5-min break in the sitting position, automatic sphygmomanometers (Omron HEM-7118, Japan) were utilized to measure the BP two times at 1-min intervals. The mean value was calculated as the final BP value. BP was measured three times in the following order: before saline loading, 2 h after saline loading (acute salt loading process), and 2 h after oral furosemide intake (diuresis shrinkage process). SBP and DBP were measured. Mean arterial pressure (MAP) was computed according to the equation MAP = (SBP + 2 × DBP)/3 (Liu et al., 2017). Subjects with an increase in the MAP of at least 5 mmHg after salt loading and/or a decrease of more than 10 mmHg after diuresis shrinkage were considered SS; otherwise, they were considered salt-resistant (SR; Liu et al., 1998).

### Candidate Gene Selection and Single-Nucleotide Polymorphism Genotyping

Utilizing knowledge of their underlying biology, a literature search strategy, and a previous genetic association study, a set of candidate genes was obtained. A total of 23 candidate genes related to SSBP were included in this study depending on their presumed roles in multiple pathways (the renin-angiotensin-aldosterone system, ion, and water channels, transporters and exchangers, the endothelial system, the apelin-APJ system, the sympathetic nervous system, intracellular messengers, and the kallikrein-kinin system; for more details, see Table 1). References of studies utilized to identify the genes can be found in the Supplementary Material. For each gene, SNPs were selected based on previously reported variants. A total of 29 SNPs identified from candidate gene studies and GWAS studies were genotyped. The primer DNA sequences are shown in Supplementary Table 2. Using the QIAamp DNA Blood Mini Kit (TIANGEN BIOTECH (BEIJING) CO., LTD, Hilden, Germany) to isolate genomic DNA from peripheral blood leukocytes according to the manual provided by the manufacturer. Genomic DNA was isolated from 200 μl of a suspension of EDTA-anticoagulated peripheral blood leukocytes using the Magnetic Beads Whole Blood Genomic DNA Extraction Kit with an automatic nucleic acid extraction apparatus (BioTeke, Beijing, China). The concentration and purity of the isolated DNA were tested using the NanoDrop 2000 spectrophotometer (Thermo Fisher Scientific, Waltham, MA, United States), according to the manual provided by the manufacturer. If the value of OD260/OD280 was between 1.7 and 2.0, and the DNA concentration was >10 ng μl^–1^, the result was considered reliable. SNPs were genotyped using the high-throughput sequencing method with the Sequenom Mass ARRAY Platform (Sequenom, San Diego, CA, United States). The quality of genotyping was controlled using blinded blood duplicates.

**TABLE 1:** Demographic and clinical characteristics of participants.

Characteristics	All subjects (*n* = 2,057)			Hypertension (*n* = 1,061)			Normotensive (*n* = 996)		
	SS (*n* = 583)	SR (*n* = 1,474)	*P-*value	SS (*n* = 302)	SR (*n* = 759)	*P*-value	SS (*n* = 281)	SR (*n* = 715)	*P-*value
Sex (male, %)	175 (30.0)	381 (25.8)	0.055	89 (29.5)	225 (29.6)	0.955	86 (30.6)	156 (21.8)	**0.004**
Age (years)	59.00 (53.00–63.00)	59.00 (54.00–64.00)	0.750	61.00 (56.00–64.00)	60.00 (54.00–64.00)	0.064	57.46 (51.69–62.00)	59.00 (53.00–63.00)	**0.014**
BMI (kg/m^2^)	25.92 (24.06–28.03)	25.92 (23.78–28.20)	0.842	26.73 (24.89–28.83)	26.87 (24.80–29.22)	0.778	25.20 (23.41–26.64)	25.15 (22.94–27.04)	0.639
WHR	0.89 (0.86–0.93)	0.89 (0.85–0.93)	0.533	0.90 (0.86–0.94)	0.89 (0.86–0.94)	0.251	0.89 (0.85–0.92)	0.88 (0.84–0.91)	**0.020**
FBG (mmol/L)	5.55 (5.01–6.44)	5.38 (4.98–6.09)	**0.003**	5.81 (5.10–6.76)	5.54 (5.05–6.37)	**0.020**	5.37 (4.96–6.00)	5.27 (4.91–5.81)	**0.048**
TG (mmol/L)	1.53 (1.09–2.42)	1.64 (1.16–2.49)	**0.044**	1.70 (1.15–2.71)	1.65 (1.18–2.47)	0.628	1.45 (1.02–2.13)	1.63 (1.12–2.53)	**0.001**
TC (mmol/L)	5.07 (4.35–5.76)	4.99 (4.34–5.66)	0.265	4.93 (4.20–5.66)	5.01 (4.20–5.70)	0.751	5.01 (4.42–5.67)	5.12 (4.50–5.80)	0.177
HDL-C (mmol/L)	1.46 (1.13–2.60)	1.44 (1.13–2.33)	0.490	1.48 (1.11–2.52)	1.48 (1.14–2.30)	0.909	1.42 (1.15–2.63)	1.37 (1.12–2.33)	0.231
LDL-C (mmol/L)	2.04 (1.47–2.83)	2.16 (1.49–2.87)	0.377	1.91 (1.32–2.93)	1.99 (1.37–2.85)	0.712	2.19 (1.59–2.80)	2.25 (1.57–2.87)	0.486
Average total walking time (minutes, per day)	60.00 (20.00–92.50)	60.00 (20.00–90.00)	0.637	40.00 (0.00–77.50)	60.00 (00.00–90.00)	0.447	60.00 (30.00–120.00)	60.00 (30.00–120.00)	0.944
Self-reported salt-eating habit (no., %)									
Eat sparingly or on average	338 (60.6)	923 (64.9)	**0.042**	175 (63.2)	441 (62.3)	0.826	163 (58.0)	482 (67.4)	**0.006**
Eat salty	220 (39.4)	500 (35.1)		102 (36.8)	267 (37.7)		118 (42.0)	233 (32.6)	
Hypertension	1,069 (51.96)	997 (48.47)	0.900	–	–	–	–	–	–
Baseline (mm Hg, mean ± SD)									
SBP	131.00 (118.00–144.00)	136.50 (124.50–148.50)	<**0.001**	138.50 (127.50–149.13)	143.17 (133.00–153.50)	<**0.001**	121.50 (113.00–134.25)	128.00 (117.00–139.50)	<**0.001**
DBP	76.00 (69.00–83.50)	79.00 (72.50–87.00)	<**0.001**	79.50 (72.88–87.00)	82.50 (77.00–90.00)	<**0.001**	72.00 (65.75–78.00)	75.50 (69.00–82.00)	<**0.001**
MAP	94.50 (86.50–102.67)	98.67 (90.83–106.83)	<**0.001**	99.67 (91.63–107.17)	103.00 (96.67–110.33)	<**0.001**	89.00 (81.83–96.75)	92.83 (86.00–101.17)	<**0.001**
Response to acute salt loading (mm Hg, mean ± SD)									
SBP	5.00 (0.00–10.00)	−7.50 (−13.50 to −2.00)	<**0.001**	6.00 (0.50–11.50)	−7.00 (−1.00 to −13.50)	<**0.001**	4.50 (−0.50 to 8.50)	−8.00 (−13.00 to −3.00)	<**0.001**
DBP	0.50 (−2.00 to 4.00)	−7.00 (−10.50 to −4.00)	<**0.001**	0.50 (−2.08 to 4.00)	−6.50 (−11.00 to −3.50)	<**0.001**	1.00 (−1.50 to 4.25)	−7.50 (−10.5 to −4.50)	<**0.001**
MAP	7.66 (5.99–10.83)	−1.84 (−5.84 to 1.49)	<**0.001**	7.49 (5.83–10.83)	−2.42 (−7.17 to −0.99)	<**0.001**	7.99 (6.16–10.99)	−1.17 (−4.51 to 1.83)	<**0.001**
Response to diuresis shrinkage (mm Hg, mean ± SD)									
SBP	−9.00 (−16.50 to −1.50)	−2.00 (−7.50 to 4.50)	<**0.001**	−10.50 (−18.50 to −2.50)	−2.50 (−8.50 to 4.00)	<**0.001**	−7.50 (−15.00 to −0.5)	−1.50 (−7.00 to 4.50)	<**0.001**
DBP	−1.50 (−6.50 to 2.50)	3.50 (0.00–7.50)	<**0.001**	−2.00 (−7.13 to 2.50)	3.00 (−0.50 to 7.50)	<**0.001**	−1.50 (−5.50 to 2.50)	4.00 (0.50–8.00)	<**0.001**
MAP	−3.83 (−10.00 to 1.00)	1.67 (−2.17 to 5.83)	<**0.001**	−4.58 (−11.00 to 0.71)	1.33 (−2.67 to 5.50)	<**0.001**	−2.67 (−8.42 to 1.17)	2.17 (−1.17 to 6.33)	<**0.001**

*SS, salt sensitivity; SR, salt resistance; BMI, body mass index; WHR, waist-hip ratio; FBG, fasting blood-glucose; TC, total cholesterol; TG, triglyceride; HDL-C, high density lipoprotein cholesterol; LDL-C, low density lipoprotein cholesterol; SBP, systolic blood pressure; DBP, diastolic blood pressure; MAP, mean arterial pressure.*

*P-value < 0.05 are bolded.*

### Salt Sensitivity of Blood Pressure Risk Factors

Fasting peripheral venous blood samples were collected to measure the biochemical parameters. Fasting blood glucose (FBG) was measured via the hexokinase/glucose-6-phosphate dehydrogenase method. The total cholesterol (TC), triglyceride (TG), low-density lipoprotein cholesterol (LDL-C), and high-density lipoprotein cholesterol (HDL-C) concentrations were tested by enzymatic methods. The waist/hip ratio (WHR) was defined as waist circumference (cm) divided by hip circumference (cm). Potential risk factors (FBG, TC, TG, LDL-C, HDL-C, and WHR) were selected in the mediation analysis based on the previous literature on SSBP (Yazdanpanah et al., 2007; Ochoa et al., 2008; Chen et al., 2009; Niemiec et al., 2011; Ding et al., 2018).

### Phenome-Wide Association Analysis

Phenome-wide association analysis (Phewas) aims to analyze the causal linkage between known genetic variants and any type of trait. We detected the Phewas results for SSBP-related SNPs within GeneATLAS 18 (2018 version). GeneATLAS was conducted on 452,264 participants and 778 phenotypes from the UK Biobank^1^.

### Expression Quantitative Trait Loci Analysis

Trait-associated SNPs are expression quantitative trait loci (eQTLs) that can regulate gene expression. We utilized the GTEx database^2^ to identify the effects of positive SNPs on the gene expression levels. The GTEx project created a large database of tissue-specific eQTLs from over 40 tissues (Consortium, 2013).

### Statistical Methods

The SSBP phenotypes were defined continuously as SBP, DBP, and MAP changes during the acute salt loading process (ΔSBP1, ΔDBP1, and ΔMAP1) and diuresis shrinkage process (ΔSBP2, ΔDBP2, and ΔMAP2), respectively, in MSAOSL-DST. For continuous phenotypes with a normal distribution, Student’s *t*-test was used to examine the differences between the SS and SR groups. The Wilcoxon rank-sum non-parametric test was used to explore the continuous variables with a non-normal distribution and the rank variables. Chi-square (χ2) analysis was used to compare the distribution of genotypic and allelic frequencies between the case and control groups and to analyze the Hardy-Weinberg equilibria. The effect of each SNP was analyzed using additive, dominant, and recessive models. Generalized linear models were utilized to account for the associations of the SNPs with SSBP and the risk factors. Multivariate logistic regression models were conducted to analyze the associations of the SNP–SNP interactions, which were estimated by ORs and 95% CIs. In our application of a mediation analysis, we considered each SNP to be the independent variable, SSBP phenotypes to be the outcome, and risk factors (WHR, TC, TG, HDL-C, LDL-C, and FBG) to be the mediator that may explain a portion of the SSBP risk. We used a two-step approach implemented in the R package “mediate” (Imai et al., 2010). The model-based causal mediation analysis was conducted in two steps. In step 1, a mediator model and an outcome model were fitted. The mediator model was a linear regression of log (risk factors) with the SNP, age, sex, and hypertension as the predictors. The outcome model was a linear regression model for SSBP phenotypes with the following covariates: SNP, log (risk factors), SNP^∗^log (risk factors) interaction term, age, sex, and hypertension. After the two models were fitted, the average causal mediation effect and average direct effect were calculated via a general algorithm (Imai et al., 2010). We performed 2,000 iterations. *P* ≤ 0.05 was considered nominally significant. To resolve multiple comparisons and control false positives and false negatives, Bonferroni correction and the Benjamini-Hochberg (BH) false discovery rate (FDR) method were conducted to perform multiple testing. The statistical analysis was performed in the IBM SPSS Statistics 24.0 software (SPSS, Chicago, IL, United States) and R software (version 3.4.4). PASS version 1.2.4 was used to calculate statistical power. In the Beijing, Chinese population, the minimal MAF of the 23 SNPs is 0.07. Through calculation, we found that with our sample size, the power to detect an OR of 1.6 is greater than 0.8.

## Results

### General Characteristics of the Study Population

A total of 2,057 participants (30% male, average age 59 years), including 583 (28.34%) SS subjects and 1,474 (71.66%) SR subjects, were included in the following analysis. The baseline characteristics of the SS and SR groups are shown in Table 1. The two groups were well matched in the distributions of age, sex, BMI, TC, HDL-C, LDL-C, physical activity, and hypertension in all subjects (*P* > 0.05). The SS group demonstrated a higher FBG level, a lower TG level, and more participants who prefer eating salty than the SR group (*P <* 0.05). In hypertensive subjects, the SS group demonstrated a higher FBG level than the SR group (*P* < 0.05). In normotensive subjects, the SS group had more female participants, a younger average age, a higher WHR and FBG, and a lower TG level, and more participants who prefer eating salty than the SR group (*P* < 0.05). Significant differences between the two groups were found in the changes in the BP response to acute salt loading and the response to diuresis shrinkage in all subjects, the hypertensive subgroup, and the normotensive subgroup (*P* < 0.05).

### Association Between Candidate Single-Nucleotide Polymorphisms and Blood Pressure Changes During Modified Sullivan’s Acute Oral Saline Load and Diuresis Shrinkage Test

**FIGURE 1:**
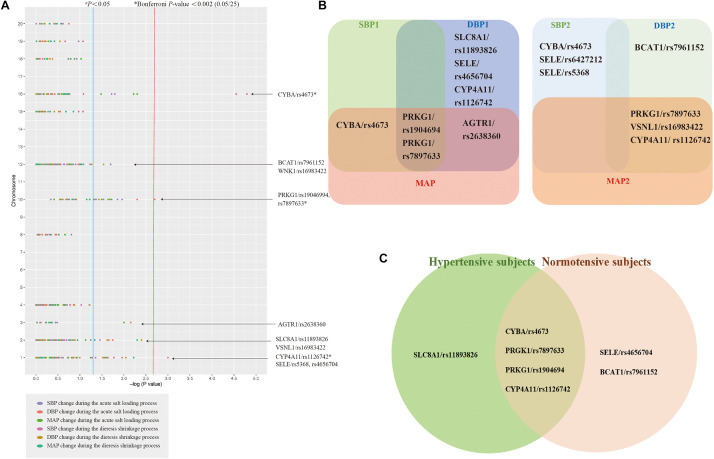
The association between the single-nucleotide polymorphisms (SNPs) and SSBP. Analysis of association between 25 SNPs in 23 genes and six phenotypes for salt sensitivity of blood pressure (SSBP) in 2,057 participants a **(A)**. The Venn diagrams comparing the identified SNPs between each SBP, DBP, MAP during acute salt loading and diuresis shrinkage in all subjects **(B)**. The Venn diagrams comparing the identified SNPs between the hypertensive subjects and normotensive subjects **(C)**. Each SNP-SSBP trait association is represented by a dot and is color coded according to the SSBP trait. ^*a*^The genes indicated down the right side of the figure are those having at least one SNP associated with one of the SSBP phenotypes with *P* < 0.05. *Bonferroni correction *P*-value < 0.002.

Detailed information on 29 candidate SNPs in 23 genes is shown in online Supplementary Table 2. All SNPs were found to be in the Hardy-Weinberg equilibrium (*P* > 0.05), except for rs1801058, rs5351, rs5516, and rs758116, which we excluded in our further analysis. Figure 1A and Supplementary Table 3 present the association between each SNP and the SBP, DBP, and MAP responses to acute salt loading and diuresis shrinkage (SSBP phenotypes) during MSAOSL-DST using univariate analysis in different genetic models (the additive, dominant, and recessive model). Eleven SNPs in eight genes (PRKG1, BCAT1, CYBA, SLC8A1, AGTR1, SELE, CYP4A11, and VSNL1) were identified to be nominally associated with one or more SSBP phenotypes. Table 2 and Figure 1B show the associations of the 11 positive SNPs with SSBP after adjusting for age, sex, FBG, TGs, physical activity, diet, and hypertension. Although 11 SNPs in eight genes were identified to be associated (*P* < 0.05) with one or more SSBP phenotypes, four SNPs (PRKG1/rs7897633 and rs1904694, CYP4A11/rs1126742, and CYBA/rs4673) displayed evidence of pleiotropy, revealing associations with three or more SSBP phenotypes in our study. After Bonferroni correction, PRKG1/rs1904694 and rs7897633, CYP4A11/rs1126742, and CYBA/rs4673 were significantly associated with at least one SSBP phenotype (*P* < 0.0007 = 0.05/25/3) after adjustment (Table 2). The PRKG1/rs1904694-G allele increased the DBP by 0.944 mmHg (*P* = 2.83E-04) in the additive model and the PRKG1/rs1904694-A allele increased the DBP by 1.351 mmHg (*P* = 4.40E-04) in the dominant model during the acute salt loading process. The CYBA/rs4673-A allele decreased the SBP by 1.652 mmHg (*P* = 1.80E-04) in the additive model and 1.850 mmHg (*P* = 3.14E-04) in the dominant model during the diuresis shrinkage process. The CYP4A11/rs1126742-C allele decreased the DBP and MAP by 5.671 mmHg (*P* = 2.90E-05) and 4.145 mmHg (*P* = 2.21E-04) in the recessive model during the diuresis shrinkage process, respectively. After the multivariate logistic regression analysis, three SNPs (PRKG1/rs7897633, CYP4A11/rs1126742, and CYBA/rs4673) still nominally associated with SS risk (Supplementary Table 4).

**TABLE 2:** Associations between SNPs and BP change after acute salt loading and diuresis shrinkage.

Genes	SNPs	EA/non-EA	Additive		Dominant		Recessive	
			β (95% CI)	*P* ^a^	β (95% CI)	*P* ^a^	β (95% CI)	*P* ^a^
Acute salt loading								
ΔSBP1^b^								
PRKG1	rs1904694	G/A	0.989 (0.153, 1.825)	**0.020**	1.313 (0.178, 2.449)	**0.023**	−1.117 (−2.848, 0.614)	0.206
PRKG1	rs7897633	A/C	0.935 (0.142, 1.728)	**0.021**	1.057 (−0.181, 2.296)	0.094	1.454 (0.095, 2.814)	**0.036**
CYBA	rs4673	A/G	0.694 (−0.229, 1.617)	0.140	1.371 (0.240, 2.501)	**0.018**	1.420 (−0.957, 3.797)	0.242
ΔDBP1^b^								
SLC8A1	rs11893826	A/G	0.621 (0.092, 1.151)	**0.022**	0.627 (−0.058, 1.311)	0.073	1.237 (0.048, 2.426)	**0.041**
PRKG1	rs1904694	G/A	0.944 (0.435, 1.453)	**2.830×10^−4^**	1.169 (0.478, 1.859)	**0.001**	1.319 (0.265, 2.372)	**0.014**
PRKG1	rs7897633	A/C	0.799 (0.317, 1.281)	**0.001**	1.351 (0.598, 2.104)	**4.440×10^−4^**	0.719 (−0.110, 1.548)	0.089
AGTR1	rs2638360	G/A	0.210 (−0.604, 1.023)	0.613	0.048 (−0.872, 0.968)	0.919	3.032 (0.185, 5.879)	**0.007**
SELE	rs4656704	G/A	0.478 (0.044, 0.913)	**0.031**	0.724 (0.018, 1.431)	**0.044**	0.490 (−0.458, 1.438)	0.311
CYP4A11	rs1126742	C/T	0.228 (−0.367, 0.822)	0.453	0.137 (−0.575, 0.848)	0.707	2.457 (0.821, 4.092)	**0.003**
ΔMAP1^b^								
PRKG1	rs1904694	G/A	0.930 (0.362, 1.498)	**0.001**	1.189 (0.418, 1.960)	**0.003**	1.211 (0.035, 2.388)	**0.044**
PRKG1	rs7897633	A/C	0.731 (0.191, 1.270)	**0.008**	1.127 (0.285, 1.969)	**0.009**	0.788 (−0.137, 1.714)	0.095
AGTR1	rs2638360	G/A	0.363 (−0.533, 1.259)	0.427	0.105 (−0.909, 1.119)	0.893	3.446 (0.310, 6.582)	**0.031**
CYBA	rs4673	A/G	0.636 (0.005, 1.266)	**0.048**	0.562 (−0.212, 1.335)	0.155	1.742 (0.119, 3.364)	**0.035**
Diuresis shrinkage								
ΔSBP2^b^								
CYBA	rs4673	A/G	−1.652 (−2.514, −0.789)	**1.800×10^−4^**	−1.850 (−3.009, −0.891)	**3.140×10^−4^**	−2.364 (−4.594, −0.134)	**0.038**
SELE	rs6427212	A/G	−0.368 (−1.180, 0.445)	0.374	−0.876 (−1.891, −0.030)	**0.050**	−1.173 (−0.651, 2.997)	0.207
SELE	rs5368	A/G	−0.857 (−1.615, −0.098)	**0.027**	0.667 (−0.389, 1.724)	0.216	1.132 (−0.892, 3.156)	0.273
ΔDBP2^b^								
PRKG1	rs7897633	A/C	−0.943 (−1.731, −0.155)	**0.019**	−2.041 (−3.269, −0.813)	**0.001**	−0.310 (−1.663, 1.043)	0.653
BCAT1	rs7961152	C/A	−1.136 (−2.368, 0.096)	0.071	−1.893 (−3.638, −0.148)	**0.034**	0.416 (−4.211, 5.043)	0.860
VSNL1	rs16983422	A/G	−1.648 (−0.191, −3.104)	**0.027**	−2.368 (−4.029, −0.743)	**0.004**	2.648 (−2.355, 7.651)	0.299
CYP4A11	rs1126742	C/T	−1.456 (−2.421, −0.492)	**0.003**	−1.018 (−0.139, 2.174)	0.085	−5.671 (−8.325, −3.018)	**2.900×10^−5^**
ΔMAP2^b^								
PRKG1	rs7897633	A/C	−0.892 (−1.544, −0.241)	**0.007**	−1.802 (−2.817, −0.786)	**0.001**	−0.450 (−1.569, 0.670)	0.431
VSNL1	rs16983422	A/G	−1.151 (−2.243, −0.058)	**0.039**	−0.708 (−1.776, 0.360)	0.194	−2.846 (−6.096, 0.405)	0.086
CYP4A11	rs1126742	C/T	−1.103 (−1.900, −0.305)	**0.007**	−0.798 (−1.75, 0.1584)	0.102	−4.145 (−6.341, −1.950)	**2.210×10^−4^**

*BP, blood pressure; SNP, single-nucleotide polymorphism; EA, effect allele; non-EA, non-effect allele.*

*^*a*^Adjusted for age, gender, TG, FBG, WHR, salt-eating habit, daily walking times, and hypertension.*

*^*b*^ΔSBP1, ΔDBP1, and ΔMAP1 were defined as the BP after acute salt loading for 2 h minus BP at baseline; ΔSBP2, ΔDBP2, and ΔMAP2 were defined as the BP after taking oral furosemide for 2 h minus BP before taking oral furosemide.*

P-value < 0.05 are bolded; *Bonferroni corrected *P*-value < 0.0007 = 0.05/3/25.

### Association Analysis Between Single-Nucleotide Polymorphisms and Blood Pressure Changes During Modified Sullivan’s Acute Oral Saline Load and Diuresis Shrinkage Test in Hypertensive Subjects and Normotensive Subjects

Further stratified analysis was conducted to explore the relationship of the above mentioned 11 positive SNPs and SSBP phenotypes in hypertensive subjects and normotensive subjects. As shown in Table 3 and Figure 1C, for hypertensive subjects, the CYBA/rs4673 was significantly associated with MAP elevation under the additive model (β = 1.460, *P* < 0.0015 = 0.05/11/3) during the acute salt loading process and SBP reduction under the additive model (β = −2.524, *P* < 0.0015) and dominant model (β = −2.843, *P* < 0.0015) during the diuresis shrinkage process after Bonferroni correction. For normotensive subjects, the CYP4A11/rs1126742 was significantly associated with DBP reduction under the recessive model (β = −8.365, *P* < 0.0015) and MAP reduction under the recessive model (β = −5.622, *P* < 0.0015) during the diuresis shrinkage process.

**TABLE 3:** Associations between SNPs and BP change after acute salt loading and diuresis shrinkage stratified by hypertension status.

Gene/SNP	Hypertensive subjects						Normotensive subjects					
	Additive model		Dominant model		Recessive model		Additive model		Dominant model	Recessive model		
	β (95% CI)	*P* ^*a*^	β (95% CI)	*P* ^*a*^	β (95% CI)	*P* ^*a*^	β (95% CI)	*P* ^*a*^	β (95% CI)	*P* ^*a*^	β (95% CI)	*P* ^*a*^
Acute salt loading												
ΔSBP1												
PRKG1/rs1904694	1.518 (0.224, 2.811)	**0.022**	1.585 (−0.188, 3.357)	0.080	2.860 (0.198, 5.522)	0.035	0.484 (−0.591, 1.560)	0.377	1.010 (−0.464, 2.483)	0.179	−0.599 (−2.824, 1.626)	0.597
PRKG1/rs7897633	1.298 (0.106, 2.490)	**0.033**	0.939 (−0.999, 2.877)	0.342	2.644 (0.642, 4.647)	**0.010**	0.491 (−0.555, 1.536)	0.357	0.988 (−0.601, 2.577)	0.223	−0.048 (−1.884, 1.787)	0.959
CYBA/rs4673	0.472 (−0.992, 1.935)	0.527	1.083 (0.681, −2.848)	0.228	1.915 (−1.962, 5.793)	0.333	0.825 (−0.334, 1.985)	0.163	1.565 (0.122, 3.009)	0.034	1.133 (−1.766, 4.031)	0.443
ΔDBP1												
SLC8A1/rs11893826	0.781 (0.043, 1.518)	**0.038**	0.920 (−0.049, 1.889)	0.063	1.162 (−0.441, 2.764)	0.155	0.457 (−0.313, 1.228)	0.244	0.366 (−0.610, 1.342)	0.462	1.254 (−0.546, 3.054)	0.172
PRKG1/rs1904694	1.180 (0.462, 1.898)	**0.001**	1.269 (0.291, 2.246)	**0.011**	2.080 (0.600, 3.559)	**0.006**	0.731 (0.002, 1.460)	**0.049**	1.114 (0.125, 2.103)	**0.027**	0.538 (−0.974, 2.049)	0.485
PRKG1/rs7897633	1.033 (0.369, 1.697)	**0.002**	1.573 (0.503, 2.643)	**0.004**	1.208 (0.088, 2.329)	**0.035**	0.564 (−0.144, 1.272)	0.118	1.192 (0.126, 2.258)	**0.028**	0.123 (−1.121, 1.368)	0.846
AGTR1/rs2638360	−0.002 (−1.149, 1.145)	0.997	−0.219 (−1.542, 1.105)	0.746	1.738 (−2.013, 5.490)	0.363	0.404 (−0.765, 1.573)	0.497	0.092 (−1.205, 1.389)	0.889	4.783 (0.340, 9.226)	**0.035**
SELE/rs4656704	0.088 (−0.784, 0.608)	0.805	0.233 (−0.753, 1.220)	0.642	−0.108 (−1.461, 1.245)	0.875	0.904 (0.202, 1.606)	**0.012**	1.245 (0.225, 2.264)	**0.017**	1.137 (−0.203, 2.477)	0.096
CYP4A11/rs1126742	0.242 (−0.617, 1.102)	0.580	0.249 (−0.759, 1.258)	0.628	3.528 (1.060, 5.996)	**0.005**	0.197 (−0.633, 1.026)	0.641	0.057 (−0.956, 1.069)	0.913	1.654 (−0.545, 3.852)	0.140
ΔMAP1												
PRKG1/rs1904694	1.338 (0.492, 2.184)	**0.002**	1.447 (0.296, 2.598)	**0.014**	2.336 (0.594, 4.079)	0.009	0.649 (−0.099, 1.396)	0.089	1.126 (0.113, 2.138)	**0.029**	0.159 (−1.390, 1.708)	0.841
PRKG1/rs7897633	1.058 (0.275, 1.841)	**0.008**	1.201 (−0.062, 2.465)	0.062	1.685 (0.367, 3.002)	**0.012**	0.539 (−0.185, 1.264)	0.144	1.179 (0.088, 2.270)	**0.034**	0.066 (−1.207, 1.339)	0.919
AGTR1/rs2638360	0.525 (−0.816, 1.865)	0.442	0.302 (−1.245, 1.849)	0.702	3.192 (−1.191, 7.574)	0.153	0.230 (−0.950, 1.410)	0.703	−0.095 (−1.403, 1.214)	0.887	4.452 (−0.033, 8.937)	0.052
CYBA/rs4673	1.460 (0.668, 2.253)	**3.140×10^−4^**	1.666 (0.512, 2.821)	**0.005**	2.619 (0.700, 4.537)	**0.008**	−0.214 (−0.932, 0.504)	0.559	−0.667 (−1.683, 0.348)	0.197	0.861 (−0.980, 2.703)	0.359
Diuresis shrinkage												
ΔSBP2												
CYBA/rs4673	−2.524 (−3.661, −1.387)	**1.500×10^−4^**	−2.843 (−4.434, −1.252)	**4.780×10^−4^**	−2.987 (−5.754, −0.220)	**0.034**	−1.052 (−2.086, −0.017)	**0.046**	−1.230 (−2.639, 0.180)	0.087	−1.696 (−4.356, 0.964)	0.211
SELE/rs6427212	−0.933 (−2.041, 0.176)	0.099	−0.304 (−1.888, 1.281)	0.707	−1.146 (−4.028, 1.736)	0.435	−0.473 (−1.44, 1.496)	0.339	−0.129 (−0.719, 1.812)	0.857	−1.243 (−3.554, 1.069)	0.292
SELE/rs5368	−0.771 (−2.049, 0.506)	0.236	−0.914 (−2.491, 0.664)	0.256	−1.079 (−4.283, 2.124)	0.509	−0.449 (−1.547, 0.649)	0.423	−0.369 (−1.777, 1.039)	0.607	−1.228 (−3.797, 1.341)	0.348
ΔDBP2												
PRKG1/rs7897633	−0.467 (−1.151, 0.217)	0.180	−1.071 (−2.171, 0.030)	0.057	−0.155 (−1.307, 0.997)	0.792	−1.446 (−2.908, 0.015)	0.052	−3.062 (−5.261, −0.863)	**0.006**	−0.311 (−2.882, 2.260)	0.812
BCAT1/rs7961152	−0.286 (−1.370, 0.797)	0.604	−0.129 (−1.333, 1.074)	0.833	2.469 (−1.526, 6.464)	0.225	−2.081 (−4.267, 0.231)	0.079	−2.574 (−5.048, 0.100)	**0.041**	−1.521 (−10.090, 7.047)	0.728
VSNL1/rs16983422	−0.056 (−1.050, 0.938)	0.912	−0.376 (1.514, 0.762)	0.517	−2.474 (−5.72, 3.775)	0.135	−0.960 (−3.074, 1.153)	0.373	−1.443 (−3.793, 0.908)	0.229	−2.741 (−10.463, 4.982)	0.486
CYP4A11/rs1126742	−0.690 (−1.555, 0.176)	0.118	−0.569 (1.586, 0.448)	0.272	−2.308 (4.806, 0.189)	0.070	−2.049 (−3.751, 0.346)	**0.018**	−1.283 (−3.367, 0.801)	0.227	−8.365 (−12.861, −3.869)	**2.780×10^−4^**
ΔMAP2												
PRKG1/rs7897633	−0.440 (−1.158, 0.278)	0.229	−0.831 (−1.987, 0.326)	0.159	−0.340 (−1.550, 0.869)	0.581	−1.357 (−2.467, −0.246)	**0.017**	−2.802 (−4.472, −1.133)	**0.001**	−0.387 (−2.343, 1.570)	0.698
VSNL1/rs16983422	−0.161 (−1.203, 0.880)	0.761	−0.438 (−1.630, 0.754)	0.471	−1.854 (−5.262, 1.554)	0.286	−0.588 (−2.197, 1.022)	0.474	−1.061 (−2.851, 0.728)	0.245	−3.601 (−9.476, 2.274)	0.229
CYP4A11/rs1126742	−0.696 (−1.607, 0.216)	0.134	0.580 (−0.491, 1.651)	0.288	−2.292 (−4.923, 0.339)	0.292	−1.363 (−2.660, −0.067)	**0.039**	0.842 (−0.744, 2.428)	0.298	−5.622 (−9.050, −2.194)	0.001^*^

^*a*^Adjusted for age, gender, TG, FBG, WHR, salt-eating habit, daily walking times, and hypertension; *P-*value < 0.05 are bolded, *Bonferroni corrected *P*-value < 0.0015 = 0.05/3/11.

### Genetic Interaction Between CYBA/rs4673 and AGTR1/rs2638360 on the Risk of Salt Sensitivity

**FIGURE 2:**
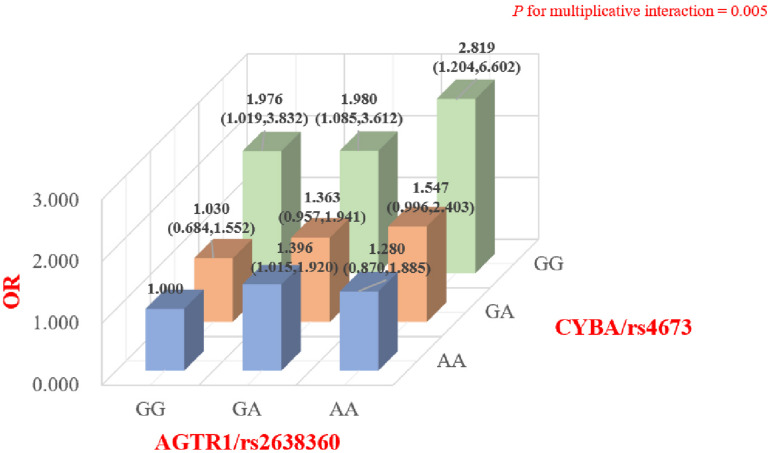
The interaction between CYBA/rs4673 and AGTR1/rs2638360.

The online software STRING was used to analyze the potential gene interactions among the abovementioned positive SNPs. Potential gene co-expression interactions between CYBA and AGTR1 were predicted by STRING. As shown in Figure 2 and Supplementary Table 5, the genetic interaction analysis showed that there was a significant interaction between CYBA/rs4673 and AGTR1/rs2638360 in the multiplicative interaction on the risk of SS (*P* = 0.004). We further tested our hypothesis by stratified analysis to determine the joint genotypic effect of the rs4673 and rs2638360 genotypes. We found that compared with those subjects without risk alleles at both loci (rs4673-GG, rs2638360-AA), subjects carrying the rs4673-A allele and/or rs2638360-G allele exhibited a higher SS risk, and the OR was strongest in individuals homozygous for the risk alleles at both loci (rs4673 AA, rs2638360 GG; OR = 2.819, *P* = 0.017). Collectively, these results indicated that carriers of both rs4673 and rs2638360 risk alleles have a higher genetic predisposition to SS risk, which is likely due to the potential gene co-expression interaction between CYBA and AGTR1.

### Cross Phenotype Analysis

GeneATLAS showed that four SSBP-related SNPs (CYBA/rs4673, SLC8A1/rs11893826, SELE/rs4656704, and rs5368) were associated with anthropometric phenotypes (BMI, waist circumference, hip circumference) and/or hypertension and/or routine blood test-associated index (Supplementary Table 6). These findings imply possible covariations between SSBP, risk factors, and hypertension. Regarding pleiotropy with body fat and hypertension, we observed that CYBA/rs4673 was significantly associated with the hip circumference in our subjects (*P* < 0.004 = 0.05/4/3) after Bonferroni correction.

### Mediating Effect of the Risk Factors on the Relationship Between Single-Nucleotide Polymorphisms and Salt Sensitivity of Blood Pressure

Several risk factors (WHR, TC, TG, HDL-C, LDL-C, and FBG) were significantly associated with SSBP (Supplementary Table 7). The association between each positive SNP and the risk factors was analyzed by the correlation method (Supplementary Table 8). CYBA/rs4673 was significantly related to TC, HDL-C, and LDL-C (FDR-*P* < 0.05); PRKG1/rs7897633 was nominally related to TG (*P* = 0.003) and FBG (*P* < 0.05); AGTR1/rs2638360 was significantly associated with TG (FDR-*P* < 0.05); SELE/rs5368 was nominally associated with FBG (*P* = 0.025); VSNL1/rs16983422 was nominally significantly associated with TG (*P* = 0.014); PRKG1/rs1904694 was nominally associated with FBG (*P* = 0.038); and CYP4A11/rs1126742 was nominally associated with TC and TG (*P* < 0.05).

**FIGURE 3:**
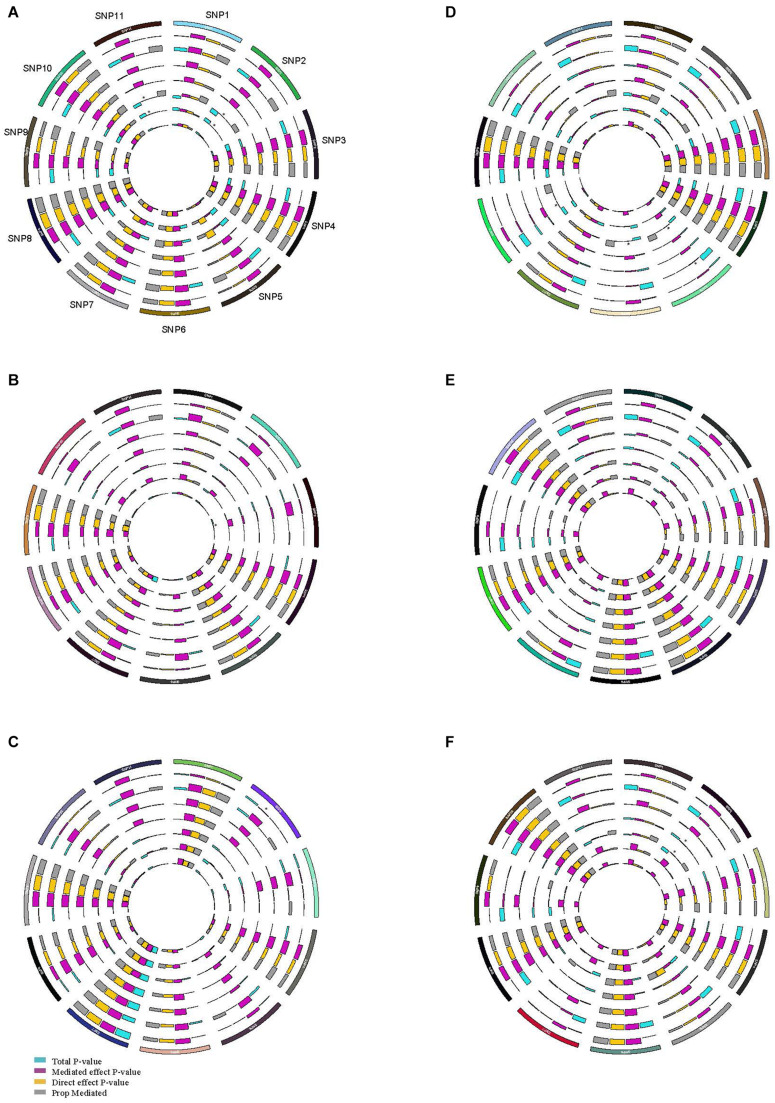
Circos plot showing the mediation model of the SNP, risk factors, and SSBP phenotypes. SNP, risk factors, and SBP increase during acute salt loading **(A)**; SNP, risk factors, and DBP increase during acute salt loading **(B)**; SNP, risk factors, and MAP increase during acute salt loading **(C)**; SNP, risk factors, and SBP decease during diuresis shrinkage **(D)**; SNP, risk factors, and DBP decease during diuresis shrinkage **(E)**; SNP, risk factors, and MAP decease during diuresis shrinkage **(F)**; SNP1 BCAT1/rs7961152, SNP2 PRKG1/rs7897633, SNP3 SLC8A1/rs11893826, SNP4 AGTR1/rs2638360, SNP5 CYBA/rs4673, SNP6 SELE/rs6427212, SNP7 CYP4A11/rs1126742, SNP8 SELE/rs5368, SNP9 VSNL1/rs16983422, SNP10 SELE/rs4656704, SNP11 PRKG1/rs1904694; The outer layer to the inner of ring were BMI, WHR, TC, TG, HDLC, LDLC, FBG, respectively. Purple indicates the mediated effect *p*-value; orange indicates the direct effect *p*-value; blue indicates the total *p*-value; grey indicates the mediation proportion; *mediated effect *P* < 0.05.

In the gene-risk factor-SSBP pathways, mediation models were set up, with the risk factors for SSBP as a mediator, to test the direct and indirect effects of the SNPs on SSBP, and the results are illustrated in Figure 3. We found significant mediating effects in the WHR as a mediator, from CYBA/rs4673 (*P* = 0.042) to SBP changes during the diuresis shrinkage process, and the direct effect was also significant (*P* < 0.000), mediating 73.2%. We found significant mediating effects of TG, from PRKG1/rs7897633 (*P* = 0.048) to MAP changes during the diuresis shrinkage process, and the direct effect was also significant (*P* = 0.026), with a mediating proportion of 24.5%. We found significant mediating effects of BMI, from PRKG1/rs7897633 (*P* = 0.045) to MAP change during the diuresis shrinkage process, and the direct effect was also significant (*P* = 0.007), with a mediating proportion of 20.3%. In PRKG1/rs7897633-SSBP path model, the mediating effect was significant from rs7897633 to SBP changes during the acute salt loading process through LDL-C (*P* = 0.006), and the direct effect was also significant (*P* = 0.006), with a mediating proportion of 46.5%. In PRKG1/rs1904694 in the SSBP path model, the mediating effect was significant from rs1904694 to SBP changes during the acute salt loading process through LDL-C (*P* = 0.022), and the direct effect was also significant (*P* = 0.020), with a mediating proportion of 45.8%. In PRKG1/rs7897633 in the SS path model, the mediating effect was significant from rs7897633 to MAP changes during the acute salt loading process through FBG (*P* = 0.016), and the direct effect was also significant (*P* = 0.004), with a mediating proportion of 20.5%. We also found significant mediating effects of FBG, from SLC8A1/rs11893826 (*P* = 0.044) to DBP changes during the acute salt loading process, and the direct effect was also significant (*P* = 0.006), with a mediating proportion of 14.4%. In PRKG1/rs7897633 and rs1904694, CYBA/rs4673, SELE/rs6427212, and rs5368 in the SSBP path model, the mediating effect was significant from the SNP to BP change during MSAOSL-DST through HDL-C (*P* < 0.05), and the direct effect was also significant (*P* < 0.05), but the total effect might be suppressed by SNPs, leading to a change in HDL-C levels (*P* > 0.05).

### Expression Quantitative Trait Loci Analysis

To support the associations between the identified 11 SNPs and SS risk, we conducted the eQTL analysis to evaluate the relationship between the SNPs and their corresponding mRNA expression levels. Using the GTEx database, we found that five SNPs (CYBA/rs4673, PRKG1/rs7897633 and rs1904694, SELE/rs4656704 and AGTR1/rs2638360) showed cis-eQTL effects on the gene expression levels (Supplementary Figure 1). rs4673 was significantly associated with the gene expression of CYBA in three types of human tissues: “Artery – Aorta” (*P* = 7.4E-8), “Cells – Cultured fibroblasts” (*P* = 5.6E-11), and “Nerve – Tibial” (*P* = 5.10E-06); rs1904694 and rs7897633 were significantly associated with the gene expression of PRKG1 in several types of human brain tissues (*P* < 3.90E-05); rs4656704 was significantly associated with the gene expression of SELE in a human liver (*P* = 1.7E-8); rs2638360 was significantly associated with the gene expression of AGTR1 in eight types of human tissues: “Cells-Cultured fibroblasts”(1.0E-33), “Nerve-Tibial”(5.4E-12),“Adipose-Subcutaneous”(3.1E-8),“Esop hagus-Muscularis”(1.5E-7),“Colon-Sigmoid”(7.10E-06), “Musc le-Skeletal”(1.80E-05), “Heart-Left Ventricle”(4.10E-05), and “Esophagus-Gastroesophageal Junction”(4.40E-05). The anno tated genes for the SSBP-related SNPs were mainly expressed in whole blood, arteries, adipose tissue, etc (Supplementary Figure 2).

## Discussion

In our study, we reviewed the literature to select candidate genes based on the biological knowledge of relevant pathways involved in SSBP and sought to validate the role of polymorphisms in 23 candidate genes involved in seven potential pathways in explaining the genetic mechanism of SSBP in the Han Chinese population. Our study further investigated whether individuals with different genetic predispositions influence SSBP through intermediate phenotypes. The main results support a potential role for polymorphisms in PRKG1, CYP4A11, BCAT1, CYBA, SLC8A1, AGTR1, SELE, and VSNL1 with SSBP in all subjects. After adjusting for multiple testing, four SNPs (PRKG1/rs1904694 and rs7897633, CYP4A11/rs1126742 and CYBA/rs4673) were significantly associated with SSBP. Gene-Gene interaction analysis suggested the potential interaction of CYBA/rs4673 and AGTR1/rs2638360. Stratified analysis showed that CYBA/rs4673 was still significantly associated with SSBP in hypertensive subjects, and CYP4A11/rs1126742 was significantly associated with SSBP in normotensive subjects. The results of our cross-phenotype analyses indicated that the SSBP-related SNPs are highly intercorrelated with anthropometric phenotypes. After further mediation analysis, we found that six positive SNPs (PRKG1/rs7897633 and rs1904694, SLC8A1 rs11893826, CYBA/rs4673, SELE/rs6427212, and rs5368) affect SSBP by modulating different risk factors (BMI, WHR, TG, TC, HDL-C, LDL-C, and FBG).

In our study, we found that the CYBA gene C242T (rs4673) was significantly associated with SSBP in all subjects and hypertensive subjects. Superoxide-generating NAD(P)H is a multisubunit enzyme complex that plays an important role in the superoxide ion production in blood vessels (Schachinger et al., 2001). Increased activity of the NAD(P)H oxidase system was associated with decreased nitric oxide levels and vascular disease (Beckman et al., 1990; Banday et al., 2008), and several pieces of evidence indicate an association between impaired nitric oxide bioactivity and SSBP (Hayakawa et al., 1997; Schmidlin et al., 2011; Preston et al., 2014). The CYBA gene can encode the major subunit of NAD(P)H, and CYBA/rs4673 has been declared to be associated with NAD(P)H activity and vascular disease (Inoue et al., 1998). One study has shown that the rs4673-A allele could affect the function of the p22phox protein and increase the risk of coronary artery disease (Mazaheri et al., 2017). Castejon et al. (2006) reported that the rs4673-T allele was associated with an increased salt sensitivity in female but not in male in normotensive Hispanics. Our study suggested that rs4673 was associated with SBP after diuresis shrinkage after multiple testing, and the association existed in both women and men (data not shown), which was different from the previous study. rs4673 was nominally associated with the SBP and MAP after acute salt loading. The results of stratified analysis showed that, rs4673 was associated with the MAP during acute salt loading and SBP during diuresis shrinkage in hypertensive subjects.

The CYP4A11 gene T8590C (rs1126742) was significantly associated with SSBP in both hypertensive and normotensive subjects and had the same direction. CYP4A arachidonic acid monooxygenase oxidizes endogenous 20-HETE. CYP4A11, from the CYP4A gene family, could catalyze the metabolism of arachidonic acid to 20-HETE. Rs1126742 can decrease 20-HETE synthase activity and is associated with hypertension through a loss-of-function mechanism (Mayer et al., 2005), and inhibitors of 20-HETE increase SSH in rats (Hoagland et al., 2003). One study showed that the rs1126742-C allele was associated with SSBP in hypertensive individuals (mostly white people). In our previous small sample study (*n* = 342), we found that rs1126742 was nominally associated with SSBP in hypertensive subjects (Liu et al., 2018). In our current study, we found that rs1126742 was nominally associated with SSBP in all subjects who were Han Chinese. Interestingly, we found that rs1126742 was not only related to SSBP in hypertensive subjects (*n* = 1,067), but also related to SSBP in normotensive subjects.

Genetic variants in the PRKG1 gene were nominally associated with SSBP during the two processes (acute salt loading and diuresis shrinkage). The PRKG1 protein plays an important role in the regulation of cardiovascular function, relaxing smooth muscle, and may be associated with SSBP by affecting renal Na^+^ reabsorption (Han et al., 2019; Struk et al., 2019). Lorena Citterio conducted a GWAS in a white population with mild hypertension and found that rs7897633 in PRKG1 was associated with DBP changes after acute salt loading (Citterio et al., 2011). This relationship was validated in two previous studies in a Han Chinese population, one was our published study with a small sample size, and the other with a different salt loading procedure than that used in our study (Liu et al., 2018; Han et al., 2019). Our results validated that rs7897633 and rs1904694 in the PRKG1 gene were nominally associated with BP changes during both acute salt loading and diuresis shrinkage processes. The above findings suggest that the PRKG1 gene may affect SSBP by disturbing the renal sodium transport or circular smooth muscles.

Some candidate gene studies in RASS system have identified a few SNPs associated with SSH (Giner et al., 2000; Poch et al., 2001; Gu et al., 2010), but the results were inconsistent. Our findings indicated that AGTR1/rs2638360 was nominally associated with SSBP (DBP and MAP increases during acute salt loading), which is the same as the research results of Gu (the same Chinese population as the GenSalt study) (Gu et al., 2010); that is, rs2638360 was related to the DBP and MAP responses to dietary sodium intervention. The GenSalt Study was a family based, dietary-based, genome-wide association study in China, and it found that SNP rs16983422 (2.8 kilobases upstream of VSNL1) was marginally associated with DBP and MAP responses. Our study confirmed the association between rs16983422 and SSBP. Therefore, our results suggested that the associations between two SNPs (AGTR1/rs2638360 and VSNL1/rs16983422) and SSBP were consistent among different salt loading manners. The SELE gene is expressed in cytokine-stimulated endothelial cells and appears to participate in the pathogenesis of atherosclerosis (Liao et al., 2016). In the GenSalt study, SELE/rs5368 was significantly associated with decreased BP responses to low-sodium processes only in male. In our study, we found that rs5368 was nominally associated with SBP decreases only in female during the diuresis shrinkage process after stratification (data not shown).

While the relationships among genes, risk factors, and SSBP have been researched in some studies (Lifton et al., 2002; Doaei and Gholamalizadeh, 2014; Elijovich et al., 2016), an integrated gene-risk factor-SSBP model to explain the modulation effect of a genetic variant on the susceptibility to SSBP is lacking. In this study, mediation analysis was utilized to explore the gene-risk factor-SSBP pathway to analyze how SSBP-related SNPs act on SSBP by modulating the risk factors. Potential risk factors were selected based on previous studies of SSBP. One study showed that metabolic syndrome increased the BP response to salt loading (Chen et al., 2009), which suggested that obesity, hyperglycemia, and dyslipidemia may be the risk factors for SSBP (Elijovich et al., 2016). We conducted mediation analysis of the risk factors on the relationship between SNPs and SSBP. We tested the relationship between SSBP phenotypes and the risk factors in our participants. But after correcting for multiple comparisons, the associations between SSBP and the risk factors (WHR, TG, and FBG) turned to be nominally significant. Second, we tested the relationship between the SNPs and risk factors. But after correcting for multiple comparisons, the associations between the SNPs and risk factors (PRKG1/rs7897633-TG, PRKG1/rs7897633-FBG, SELE/rs5368-FBG, VSNL1/rs16983422-TG, PRKG1/rs1904694-FBG, CYP4A11/rs1126742-TC, CYP4A11/rs1126742-TG) turned to be nominally significant. In our study, we found that CYBA/rs4673 had an indirect effect on SSBP through the mediating effect of three main categories of lipoproteins (TC, HDL-C, and LDL-C) and WHR. CYP4A11/rs1126742 affected SSBP directly through the mediating effect of TC and TG. The CYBA gene encodes the major subunit of nicotinamide adenine dinucleotide phosphate (NADPH), and hypercholesterolemia influences vascular NADPH oxidases (Guzik et al., 2000). NADPH oxidases are the major source of vascular and renal reactive oxygen species-induced (ROS; Tamara and Rhian, 2008). Low-density lipoproteins (LDL) seemed to play an important role in the development of atherosclerosis, and the oxidative modification of LDL may be a key point in this process (Sampath et al., 2010). NADPH oxidase was found as a potential source for [O(2)](-) in the proliferation-inducing activity of oxidized LDL (OxLDL; Alexandra et al., 2000). CYP4A11 encodes a member of the cytochrome P450 superfamily of enzymes which are involved in the synthesis of cholesterol, steroids, and other lipids (Williams et al., 2010). 20-Hydroxyeicosatetraenoic acid (20-HETE) is a cytochrome P450 (CYP) 4A/4F-derived metabolite of arachidonic acid (Cheng et al., 2014). CYP4A11 promotes the formation of HEETs, which can affect lipid metabolism by modulating the gene expression (Staels and Fruchart, 2005). Increasing evidence has suggested that CYP4A11 may be involved in ROS lipid peroxidation and inflammation (Huifang et al., 2020). The above two genes may, through a ROS system, interact with lipoproteins that are indirectly associated with SSBP. SNPs in PRKG1 have an indirect effect on SSBP through the mediating effect of BMI, FBG, and LDL-C. Serine/threonine-protein kinase that acts as a key mediator of the nitric oxide (NO)/cGMP signaling pathway and GMP binding activates PRKG1, which phosphorylates serines and threonines on many cellular proteins (Franz, 2005). In *Drosophila melanogaster*, the product of the *for* gene, encoding PKG, might be involved in the regulation of food-related behaviors (Wilding, 2002), and thus, may be related to obesity. The epidemiological study provided indicated that the presence of a high number of metabolic risk factors was negatively associated with urinary cGMP excretion (Renzhe et al., 2007). The results from our data, body fat, blood glucose, and lipoproteins might mediate the cGMP signaling pathway involved in SSBP. In conclusion, our results suggested that obesity, diabetes, and dyslipidemia are mediators of the relation between SNP and SSBP. The precise mechanism linking these three conditions still needs further study.

Various methods of identifying SSBP have been developed. However, there is no standard protocol for identifying SSBP. In our study, MSAOSL-DST was used to distinguish SSBP. This method is a modification of Sullivan’s method, which has been used in many studies of the Chinese population (Mu et al., 1993; Liu et al., 2017, 2018). In the present study, compared with GenSalt studies that focus on the BP response to chronic salt intervention, our study focuses on the BP response to acute salt loading and diuresis shrinkage in northern China to measure SSBP. This study assesses SSBP by acute salt loading in a Han Chinese population in such a large sample. Another important strength of our study is that our subjects were recruited from the community, thereby reducing certain aspects of selection bias. Our research used two outcomes to explore the genetic variations and SSBP. We focused on the association between the SNP and quantitative SS traits represented by BP changes during acute salt loading and diuresis shrinkage and analyzed the association of genetic variations with qualitative traits (SS or SR) to validate the association of these SNPs with SSBP. The results we obtained using the two methods above were similar. Three (PRKG1/rs7897633, CYP4A11/rs1126742, and CYBA/rs4673) of the four positive SNPs that were significantly associated with SSBP were also nominally associated with SS risk. Another important advantage of our study is that we used mediation analysis to establish a united gene-risk factor-SSBP model to explain the indirect contribution of SNPs to SSBP. However, our study also have several limitations. First, the findings in this study need replication in an independent study to verify the generalizability of the identified associations. Second, our research is the cross-sectional design. Further study is required to study the directions of the relationship between the risk factors and SSBP. Third, functional tests performed on positive SNPs and risk factors to determine the potential biological mechanisms of how candidate genes and risk factors play roles in SSBP are needed.

In conclusion, this candidate gene study aimed to investigate the association between 29 SNPs in 23 candidate genes and SSBP in a Han Chinese population. We found that 11 SNPs in eight genes (PRKG1, CYBA, BCAT1, SLC8A1, AGTR1, SELE, CYP4A11, and VSNL1) were significantly associated with SSBP. Among them, four SNPs (PRKG1/rs1904694 and rs7897633, CYP4A11/rs1126742, and CYBA/rs4673) passed the Bonferroni correction. Stratified analysis showed that CYBA/rs4673 was still significantly associated with SSBP in hypertensive subjects, and CYP4A11/rs1126742 was significantly associated with SSBP in normotensive subjects. Furthermore, we identified the risk factors (WHR, BMI, TG, LDL-C, HDL-C, FBG) that are mediators of the relation between SNP and SSBP. Additional research on the potential biological mechanism is needed on the gene and gene-risk factors of SSBP.

## Data Availability Statement

The genotyping data for this article are not publicly available to assure patient confidentiality and participant privacy. The data that support the findings of this study are available on request from the corresponding author (zlilyepi@ccmu.edu.cn).

## Ethics Statement

The studies involving human participants were reviewed and approved by the Ethical Committee of Capital Medical University. The patients/participants provided their written informed consent to participate in this study. Written informed consent was obtained from the individual(s) for the publication of any potentially identifiable images or data included in this article.

## Author Contributions

LZ participated in the study design and reviewed the manuscript. YX participated in the data analysis and drafted the manuscript. ZL designed and performed the experiments. YX, ZL, HQ, WP, HC, XL, BL, FW, and FZ performed the cases collection. All authors contributed to the article and approved the submitted version.

## Conflict of Interest

The authors declare that the research was conducted in the absence of any commercial or financial relationships that could be construed as a potential conflict of interest.

## Publisher’s Note

All claims expressed in this article are solely those of the authors and do not necessarily represent those of their affiliated organizations, or those of the publisher, the editors and the reviewers. Any product that may be evaluated in this article, or claim that may be made by its manufacturer, is not guaranteed or endorsed by the publisher.

## Footnotes

1
http://geneatlas.roslin.ed.ac.uk/

2
http://www.gtexportal.org/
