# Supplementary material for: Candidate Gene Polymorphisms Influence the Susceptibility to Salt Sensitivity of Blood Pressure in a Han Chinese Population: Risk Factors as Mediators
Source: Front Genet. 2021 Oct 4;12:675230. doi: 10.3389/fgene.2021.675230 (PMC8521039; doi:10.3389/fgene.2021.675230)
Supplement: Supplementary file 1 [file Data_Sheet_1.docx]

**Supplementary Data**

**Supplementary Tables**

**Table S1.** Selection of candidate genes and SNPs

**Table S2.**Information of candidate SNPs included in the study

**Table S3.** Crude P value for association between SNPs and BP change

**Table S4.** Multiple logistic regression analysis of associations between 11 SNPs and SS

**Table S5.**Interaction analysis between AGTR1/rs2638360 and CYBA/rs4673

**Table S6.**Phenotypes associated with SSBP-related SNPs using GeneAtlas

**Table S7.***P* value (before/after adjusted) for association between SSBP phenotypes and risk factors

**Table S8.** Effect size (β) and *P* value for association between SNPs and risk factors

**Figure S1.** The Violin plot shows thecorrelation between SNP and gene expression in the GTEx database.

**Figure S2.** Standardized median gene expressions across 54 tissues for genes mapped to SSBP-related SNP.

**Table S1. Candidate genes**

| **Pathways** | **Gene** | **Description** | **Chr** | **dbSNP** | **Previous study** |
| --- | --- | --- | --- | --- | --- |
|  |  |  |  |  |  |
| Ion and water channels, transporters, and exschangers | SLC8A1 | solute carrier family 8 member A1 | 2 | rs434082 | 1, 2 |
|  |  |  |  | rs11893826 |  |
|  | SLC24A3 | solute carrier family 24 member 3 | 20 | rs6112470 | 2 |
|  | WNK1 | WNK lysine deficient protein kinase 1 | 12 | rs12828016 | 3, 4 |
|  |  |  |  | rs2255390 |  |
|  | SCNN1A | sodium channel epithelial 1 subunit alpha | 12 | rs11064153 | 5 |
|  | SCNN1G | sodium channel epithelial 1 subunit gamma | 16 | rs4401050 | 6 |
|  | CACNA1A | calcium voltage-gated channel subunit alpha1 A | 8 | rs8182538 | 7 |
|  | CACNA1C | calcium voltage-gated channel subunit alpha1 C | 12 | rs758116 |  |
| Renin-Angiotensin-Aldosterone System | CYP4A11 | cytochrome P450 family 4 subfamily A member 11 | 1 | rs1126742 | 8 |
|  | CYP11B2 | cytochrome P450 family 11 subfamily B member 2 | 8 | rs1799998 | 9-11 |
|  | AGTR1 | angiotensin II receptor type 1 | 3 | rs2638360 | 12 |
|  | NR3C2 | nuclear receptor subfamily 3 group C member 2 | 4 | rs7694064 | 13 |
|  |  |  |  | rs6856803 |  |
| Sympathetic nervous system | GRK4 | G protein-coupled receptor kinase 4 | 4 | rs1801058 | 14, 15 |
|  |  |  |  |  |  |
| Natriuretic peptide system | PRKG1 | protein kinase cGMP-dependent 1 | 10 | rs1904694 | 1 |
|  |  |  |  | rs7897633 |  |
| Intracellular messengers | VSNL1 | Visinin-like protein 1 | 2 | rs16983422 | 16 |
| Kallikrein–kinin system | KLK1 | kallikrein 1 | 19 | rs5516 | 17 |
| Endothelial system | SELE | selectin E | 1 | rs4656704 | 18 |
|  |  |  |  | rs6427212 |  |
|  |  |  |  | SNP95_rs5368 |  |
|  | CYBA | cytochrome b-245 alpha chain | 16 | rs4673 | 19 |
|  | EDNRB | endothelin receptor type B | 13 | rs5351 | 20 |
| Other | STK39 | serine/threonine kinase 39 | 2 | rs3754777 | 21, 22 |
|  | BCAT1 | solute carrier family 24 member 3 | 12 | rs7961152 | 21 |
|  | NR2F2-AS1 | NR2F2 antisense RNA 1 | 15 | rs2398162 | 21 |
|  | NEDD4L | NEDD4 like E3 ubiquitin protein ligase | 18 | rs4149601 | 23 |
|  | FGF5 | fibroblast growth factor 5 | 4 | rs16998073 | 21 |

Abbreviations: Chr=chromosome; dbSNP, database single nucleotide polymorphism

**Table 1 Reference**

1. Citterio, L.; Simonini, M.; Zagato, L.; Salvi, E.; Delli Carpini, S.; Lanzani, C.; Messaggio, E.; Casamassima, N.; Frau, F.; D'Avila, F., et al. Genes involved in vasoconstriction and vasodilation system affect salt-sensitive hypertension. *PLoS One* **2011**, *6*, e19620, doi:10.1371/journal.pone.0019620.

2. Liu, Z.; Qi, H.; Liu, B.; Liu, K.; Wu, J.; Cao, H.; Zhang, J.; Yan, Y.; He, Y.; Zhang, L. Genetic susceptibility to salt-sensitive hypertension in a Han Chinese population: a validation study of candidate genes. *Hypertens Res* **2017**, *40*, 876-884, doi:10.1038/hr.2017.57.

3. Liu, F.; Zheng, S.; Mu, J.; Chu, C.; Wang, L.; Wang, Y.; Xiao, H.; Wang, D.; Cao, Y.; Ren, K., et al. Common variation in with no-lysine kinase 1 (WNK1) and blood pressure responses to dietary sodium or potassium interventions- family-based association study. *Circ J* **2013**, *77*, 169-174, doi:10.1253/circj.cj-12-0900.

4. Osada, Y.; Miyauchi, R.; Goda, T.; Kasezawa, N.; Horiike, H.; Iida, M.; Sasaki, S.; Yamakawa-Kobayashi, K. Variations in the WNK1 gene modulates the effect of dietary intake of sodium and potassium on blood pressure determination. *J Hum Genet* **2009**, *54*, 474-478, doi:10.1038/jhg.2009.64.

5. Gu, X.; Gu, D.; He, J.; Rao, D.C.; Hixson, J.E.; Chen, J.; Li, J.; Huang, J.; Wu, X.; Rice, T.K., et al. Resequencing Epithelial Sodium Channel Genes Identifies Rare Variants Associated With Blood Pressure Salt-Sensitivity: The GenSalt Study. *Am J Hypertens* **2018**, *31*, 205-211, doi:10.1093/ajh/hpx169.

6. Zhao, Q.; Gu, D.; Hixson, J.E.; Liu, D.P.; Rao, D.C.; Jaquish, C.E.; Kelly, T.N.; Lu, F.; Ma, J.; Mu, J., et al. Common variants in epithelial sodium channel genes contribute to salt sensitivity of blood pressure: The GenSalt study. *Circ Cardiovasc Genet* **2011**, *4*, 375-380, doi:10.1161/CIRCGENETICS.110.958629.

7. Hu, Z.; Liu, F.; Li, M.; He, J.; Huang, J.; Rao, D.C.; Hixson, J.E.; Gu, C.; Kelly, T.N.; Chen, S., et al. Associations of Variants in the CACNA1A and CACNA1C Genes With Longitudinal Blood Pressure Changes and Hypertension Incidence: The GenSalt Study. *Am J Hypertens* **2016**, *29*, 1301-1306, doi:10.1093/ajh/hpw070.

8. Williams, J.S.; Hopkins, P.N.; Jeunemaitre, X.; Brown, N.J. CYP4A11 T8590C polymorphism, salt-sensitive hypertension, and renal blood flow. *J Hypertens* **2011**, *29*, 1913-1918, doi:10.1097/HJH.0b013e32834aa786.

9. Iwai, N.; Kajimoto, K.; Tomoike, H.; Takashima, N. Polymorphism of CYP11B2 determines salt sensitivity in Japanese. *Hypertension* **2007**, *49*, 825-831, doi:10.1161/01.HYP.0000258796.52134.26.

10. Wrona, A.; Widecka, K.; Adler, G.; Czekalski, S.; Ciechanowicz, A. [Promoter variants of aldosterone synthase gene (CYP11B2) and salt-sensitivity of blood pressure]. *Pol Arch Med Wewn* **2004**, *111*, 191-197.

11. Pamies-Andreu, E.; Ramirez-Lorca, R.; Stiefel Garcia-Junco, P.; Muniz-Grijalbo, O.; Vallejo-Maroto, I.; Garcia Morillo, S.; Miranda-Guisado, M.L.; Ortiz, J.V.; Carneado de la Fuente, J. Renin-angiotensin-aldosterone system and G-protein beta-3 subunit gene polymorphisms in salt-sensitive essential hypertension. *J Hum Hypertens* **2003**, *17*, 187-191, doi:10.1038/sj.jhh.1001534.

12. Gu, D.; Kelly, T.N.; Hixson, J.E.; Chen, J.; Liu, D.; Chen, J.C.; Rao, D.C.; Mu, J.; Ma, J.; Jaquish, C.E., et al. Genetic variants in the renin-angiotensin-aldosterone system and salt sensitivity of blood pressure. *J Hypertens* **2010**, *28*, 1210-1220.

13. He, W.J.; Li, C.; Rao, D.C.; Hixson, J.E.; Huang, J.; Cao, J.; Rice, T.K.; Shimmin, L.C.; Gu, D.; Kelly, T.N. Associations of Renin-Angiotensin-Aldosterone System Genes With Blood Pressure Changes and Hypertension Incidence. *Am J Hypertens* **2015**, *28*, 1310-1315, doi:10.1093/ajh/hpv033.

14. Sanada, H.; Yatabe, J.; Midorikawa, S.; Hashimoto, S.; Watanabe, T.; Moore, J.H.; Ritchie, M.D.; Williams, S.M.; Pezzullo, J.C.; Sasaki, M., et al. Single-nucleotide polymorphisms for diagnosis of salt-sensitive hypertension. *Clin Chem* **2006**, *52*, 352-360, doi:10.1373/clinchem.2005.059139.

15. Rayner, B.; Ramesar, R.; Steyn, K.; Levitt, N.; Lombard, C.; Charlton, K. G-protein-coupled receptor kinase 4 polymorphisms predict blood pressure response to dietary modification in Black patients with mild-to-moderate hypertension. *J Hum Hypertens* **2012**, *26*, 334-339, doi:10.1038/jhh.2011.33.

16. Mei, H.; Gu, D.; Hixson, J.E.; Rice, T.K.; Chen, J.; Shimmin, L.C.; Schwander, K.; Kelly, T.N.; Liu, D.P.; Chen, S., et al. Genome-wide linkage and positional association study of blood pressure response to dietary sodium intervention: the GenSalt Study. *Am J Epidemiol* **2012**, *176 Suppl 7*, S81-90, doi:10.1093/aje/kws290.

17. Svetkey, L.P.; Harris, E.L.; Martin, E.; Vollmer, W.M.; Meltesen, G.T.; Ricchiuti, V.; Williams, G.; Appel, L.J.; Bray, G.A.; Moore, T.J., et al. Modulation of the BP response to diet by genes in the renin-angiotensin system and the adrenergic nervous system. *Am J Hypertens* **2011**, *24*, 209-217, doi:10.1038/ajh.2010.223.

18. Defago, M.D.; Gu, D.; Hixson, J.E.; Shimmin, L.C.; Rice, T.K.; Gu, C.C.; Jaquish, C.E.; Liu, D.P.; He, J.; Kelly, T.N. Common genetic variants in the endothelial system predict blood pressure response to sodium intake: the GenSalt study. *Am J Hypertens* **2013**, *26*, 643-656, doi:10.1093/ajh/hps099.

19. Castejon, A.M.; Bracero, J.; Hoffmann, I.S.; Alfieri, A.B.; Cubeddu, L.X. NAD(P)H oxidase p22phox gene C242T polymorphism, nitric oxide production, salt sensitivity and cardiovascular risk factors in Hispanics. *J Hum Hypertens* **2006**, *20*, 772-779, doi:10.1038/sj.jhh.1002057.

20. Caprioli, J.; Mele, C.; Mossali, C.; Gallizioli, L.; Giacchetti, G.; Noris, M.; Remuzzi, G.; Benigni, A. Polymorphisms of EDNRB, ATG, and ACE genes in salt-sensitive hypertension. *Can J Physiol Pharmacol* **2008**, *86*, 505-510, doi:10.1139/Y08-045.

21. Rhee, M.Y.; Yang, S.J.; Oh, S.W.; Park, Y.; Kim, C.I.; Park, H.K.; Park, S.W.; Park, C.Y. Novel genetic variations associated with salt sensitivity in the Korean population. *Hypertens Res* **2011**, *34*, 606-611, doi:10.1038/hr.2010.278.

22. Fava, C.; Danese, E.; Montagnana, M.; Sjogren, M.; Almgren, P.; Engstrom, G.; Nilsson, P.; Hedblad, B.; Guidi, G.C.; Minuz, P., et al. Serine/threonine kinase 39 is a candidate gene for primary hypertension especially in women: results from two cohort studies in Swedes. *J Hypertens* **2011**, *29*, 484-491, doi:10.1097/HJH.0b013e328342b2c1.

23. Shindo, T.; Kurihara, H.; Maemura, K.; Kurihara, Y.; Ueda, O.; Suzuki, H.; Kuwaki, T.; Ju, K.H.; Wang, Y.; Ebihara, A., et al. Renal damage and salt-dependent hypertension in aged transgenic mice overexpressing endothelin-1. *J Mol Med (Berl)* **2002**, *80*, 105-116, doi:10.1007/s00109-001-0284-4.

**Table S2. Information of candidate SNPs included in the study**

| **dbSNP** | **Gene** | **Chr** | **Position** | **Function** | **Alleles  (Minor/Major)** | **effect allele** | **MAF^a^** | **HWE** | **Reverse primer sequence 5 '> 3'** | **Forward primer sequence 5 '> 3'** |
| --- | --- | --- | --- | --- | --- | --- | --- | --- | --- | --- |
| rs1126742 | *CYP4A11* | 1 | 47398496 | missense | G/A | G | 0.184 | 0.2 | ACGTTGGATGTAACTATCCTGGCTCTGGTG | ACGTTGGATGTGGCTGTGTTGAGCAGAACC |
| rs4656704 | *SELE* | 1 | 169688228 | intron | G/A | G | 0.34 | 0.645 | ACGTTGGATGACATGTGACTTTAGAGACGG | ACGTTGGATGAGACTTGAGCTACCTCCTTC |
| rs6427212 | *SELE* | 1 | 169689041 | - | A/G | A | 0.257 | 0.209 | ACGTTGGATGTGTGACCTCCGGAATAACAG | ACGTTGGATGAAGGTTAGGAGGATGAGAGG |
| rs5368 | *SELE* | 1 | 169696946 | missense | A/G | A | 0.238 | 0.513 | ACGTTGGATGAAGTCCTCTTGTGCCTTCAG | ACGTTGGATGTCCATTGTCCCTGAGATGTG |
| rs16983422 | *VSNL1* | 2 | 17444663 | - | G/A | G | 0.141 | 0.288 | ACGTTGGATGAATGAGACACTGTAGGTGGC | ACGTTGGATGTGTTGAGTGCACAGTGTAAG |
| rs434082 | *SLC8A1* | 2 | 40485074 | intron | C/T | T | 0.126 | 0.096 | ACGTTGGATGTTCTCCTTTCCTCCTCACTC | ACGTTGGATGAAGCATTTCCCGCAGCAAAG |
| rs11893826 | *SLC8A1* | 2 | 40564647 | intron | A/G | A | 0.354 | 0.384 | ACGTTGGATGAACAACAAACCACTCACCTC | ACGTTGGATGCATTGAGTGCCACTGAGTAG |
| rs3754777 | *STK39* | 2 | 169015914 | intron | C/T | T | 0.306 | 0.925 | ACGTTGGATGTCTCCACATTCCACTCTTCC | ACGTTGGATGACAAAAATGAGGACCAGGAG |
| rs2638360 | *AGTR1* | 3 | 148428356 | intron | G/A | G | 0.121 | 0.179 | ACGTTGGATGGTCTATGCAATGGTGAAATG | ACGTTGGATGTCCTTACTCATTACCTTCTG |
| rs1801058 | *GRK4* | 4 | 3039150 | intron | T/C | C | 0.471 | ＜0.001 | ACGTTGGATGAGAAGTCTTCATCTGCGGTG | ACGTTGGATGTAAGGACGTCCTGGATATCG |
| rs7694064 | *NR3C2* | 4 | 149083218 | intron | A/G | A | 0.248 | 0.088 | ACGTTGGATGTCAATGGCACTTGACTCCAG | ACGTTGGATGAGGCAGATTACACCTAAGGC |
| rs6856803 | *NR3C2* | 4 | 149087127 | intron | T/C | T | 0.476 | 0.8 | ACGTTGGATGGAGTTAACAGGATGCCATGC | ACGTTGGATGGGTTTGCTTTCTCTTTCCTC |
| rs16998073 | *FGF5* | 5 | 81184341 | - | A/T | T | 0.383 | 0.385 | ACGTTGGATGTTCCTTCACTCTAGAGACCG | ACGTTGGATGTCTTGCCTTCAGGACCCTTG |
| rs1799998 | *CYP11B2* | 8 | 143999600 | upstream variant 2KB | A/G | G | 0.262 | 0.085 | ACGTTGGATGGGGACTTTATCTTATCGTGAG | ACGTTGGATGGCAATGAACTAAATCTGTGG |
| rs1904694 | *PRKG1* | 10 | 52905494 | intron | G/A | G | 0.393 | 0.945 | ACGTTGGATGGTTAGATGTTAACAGATGGG | ACGTTGGATGTTTGGAAGTGGATGAACACC |
| rs7897633 | *PRKG1* | 10 | 52957721 | intron | A/C | A | 0.476 | 0.824 | ACGTTGGATGTTCACCACAGATTTGAGAGG | ACGTTGGATGCCTTTAGACAAACATTCGAC |
| rs12828016 | *WNK1* | 12 | 998365 | missense | G/T | T | 0.257 | 0.435 | ACGTTGGATGCCTCTAGAGGATCTTGATGC | ACGTTGGATGTTACACCAACCGCAGAAGTC |
| rs2255390 | *WNK1* | 12 | 999762 | intron | A/G | G | 0.466 | 0.704 | ACGTTGGATGTTACTACCAATGCTCCTCAG | ACGTTGGATGACCCGTAGTTGATTCAGGAG |
| rs758116 | *CACNA1C* | 12 | 2523256 | intron | G/A | G | 0.301 | ＜0.001 | ACGTTGGATGAGATTACGCCACTTATCCCG | ACGTTGGATGATTCAGAGTAAGACTGGCCC |
| rs11064153 | *SCNN1A* | 12 | 6488450 | intron | T/C | T | 0.403 | 0.553 | ACGTTGGATGTCTGCAGAAGACAGCAGAAC | ACGTTGGATGTTTCGTAGCATATCCCGCTC |
| rs8182538 | *CACNA1C* | 12 | 13416170 | intron | G/A | A | 0.437 | 0.533 | ACGTTGGATGGGTGCTGTTTGAATATCCCC | ACGTTGGATGGAGAAAGACCAAGGCAAAGG |
| rs7961152 | *BCAT1* | 12 | 24981611 | intron | A/C | A | 0.136 | 0.865 | ACGTTGGATGCCCAGCCAGTATACTTTTAG | ACGTTGGATGGCAAAGCATTTATTCAGTG |
| rs5351 | *EDNRB* | 13 | 78475313 | synonymous codon | T/C | C | 0.301 | ＜0.001 | ACGTTGGATGCCAATGGCAAGCAGAAATAG | ACGTTGGATGCGTAAAAATTCTCTCATCCC |
| rs2398162 | *NR2F2-AS1* | 15 | 96830550 | intron | A/G | A | 0.369 | 0.189 | ACGTTGGATGATGAAGGTGAACTCCAGCTC | ACGTTGGATGGGCTTTCCTTCTCAACCTTA |
| rs4401050 | *SCNN1G* | 16 | 23217402 | intron | C/T | T | 0.113 | 0.894 | ACGTTGGATGGATGAATGTAGCTGAGCCTG | ACGTTGGATGCTGTCTGCACCCTGTTTTAG |
| rs4673 | *CYBA* | 16 | 88713236 | missense | A/G | A | 0.073 | 0.964 | ACGTTGGATGAGCAAAGGAGTCCCGAGTG | ACGTTGGATGAACAGCTTCACCACGGCGG |
| rs4149601 | *NEDD4L* | 18 | 55816791 | synonymous codon | G/A | A | 0.209 | 0.292 | ACGTTGGATGAGGAAGGTAAAACCTCCTCC | ACGTTGGATGTCCTAAATGAGACGTCTCGC |
| rs5516 | *KLK1* | 19 | 51323473 | missense | C/G | C | 0.175 | ＜0.001 | ACGTTGGATGAAACAGGTGCTCCCCACTTC | ACGTTGGATGGCTGATACCATCACAGATGC |
| rs6112470 | *SLC24A3* | 20 | 19562579 | intron | C/T | C | 0.165 | 0.258 | ACGTTGGATGAGAGCAACCACATTTTCCTG | ACGTTGGATGGCACCCACTCTTGTTATATG |

Abbreviations: dbSNP, database SNP; Chr, chromosome; MAF, minor allele frequency; HWE, Hardy-Weinberg equilibrium.

^a^MAF was extracted from the 1000 Genomes CHBpopulation (http://www.internationalgenome.org/).

**Table S3. Crude *P* value for association between SNPs and BP change**

| **Gene** | **dbSNP** | **ΔSBP1^a^** | | | **ΔDBP1 ^a^** | | | **ΔMAP1 ^a^** | | | **ΔSBP2 ^a^** | | | **ΔDBP2 ^a^** | | | **ΔMAP2 ^a^** | | |
| --- | --- | --- | --- | --- | --- | --- | --- | --- | --- | --- | --- | --- | --- | --- | --- | --- | --- | --- | --- |
|  |  | **additive** | **dominant** | **recessive** | **additive** | **dominant** | **recessive** | **additive** | **dominant** | **recessive** | **additive** | **dominant** | **recessive** | **additive** | **dominant** | **recessive** | **additive** | **dominant** | **recessive** |
| *CYP4A11* | SNP3_rs1126742 | 0.745 | 0.368 | 0.241 | 0.418 | 0.639 | **0.001^b^** | 0.989 | 0.343 | 0.027 | 0.331 | 0.586 | 0.154 | **0.028** | 0.139 | **0.009** | **0.025** | 0.14 | **0.006** |
| *SLC8A1* | SNP6_rs11893826 | 0.651 | 0.618 | 0.883 | **0.005** | **0.022** | **0.018** | 0.074 | 0.133 | 0.160 | 0.891 | 0.749 | 0.804 | 0.267 | 0.277 | 0.545 | 0.306 | 0.29 | 0.645 |
| *FGF5* | SNP8_rs16998073 | 0.700 | 0.902 | 0.575 | 0.453 | 0.952 | 0.139 | 0.683 | 0.911 | 0.364 | 0.769 | 0.565 | 0.848 | 0.307 | 0.239 | 0.695 | 0.321 | 0.224 | 0.773 |
| *PRKG1* | SNP11_rs1904694 | **0.029** | **0.011** | **0.460** | **0.014** | **0.021** | **0.142** | 0.060 | **0.047** | 0.385 | 0.150 | 0.286 | 0.176 | 0.121 | 0.201 | 0.207 | 0.070 | 0.146 | 0.127 |
| *STK39* | SNP19_rs3754777 | 0.163 | 0.175 | 0.449 | 0.132 | 0.130 | 0.473 | 0.145 | 0.113 | 0.654 | 0.250 | 0.333 | 0.354 | 0.382 | 0.232 | 0.827 | 0.262 | 0.177 | 0.930 |
| *SLC8A1* | SNP20_rs434082 | 0.500 | 0.712 | 0.233 | 0.632 | 0.760 | 0.474 | 0.831 | 0.955 | 0.366 | 0.200 | 0.222 | 0.496 | 0.763 | 0.678 | 0.821 | 0.512 | 0.461 | 0.995 |
| *SLC24A3* | SNP23_rs6112470 | 0.817 | 0.645 | 0.645 | 0.629 | 0.984 | 0.179 | 0.653 | 0.739 | 0.633 | 0.242 | 0.359 | 0.259 | 0.484 | 0.638 | 0.388 | 0.331 | 0.488 | 0.269 |
| *PRKG1* | SNP27_rs7897633 | **0.014** | **0.043** | **0.048** | **0.005** | **0.002** | **0.177** | **0.032** | **0.021** | 0.270 | 0.129 | 0.245 | 0.194 | **0.037** | **0.023** | 0.287 | **0.019** | **0.019** | 0.178 |
| *BCAT1* | SNP28_rs7961152 | 0.083 | **0.226** | **0.020** | 0.283 | 0.437 | 0.170 | 0.449 | 0.574 | 0.359 | 0.228 | 0.442 | 0.059 | **0.054** | **0.029** | 0.393 | 0.181 | 0.091 | 0.496 |
| *WNK1* | SNP35_rs12828016 | 0.709 | 0.230 | 0.182 | 0.475 | 0.357 | 0.991 | 0.421 | 0.161 | 0.494 | 0.724 | 0.685 | 0.933 | 0.155 | 0.349 | 0.103 | 0.172 | 0.342 | 0.142 |
| *VSNL1* | SNP37_rs16983422 | 0.410 | 0.764 | 0.057 | 0.792 | 0.831 | 0.799 | 0.727 | 0.857 | 0.516 | **0.986** | **0.473** | **0.035** | **0.024** | **0.004** | 0.284 | **0.046** | **0.005** | 0.113 |
| *CYP11B2* | SNP38_rs1799998 | 0.607 | 0.870 | 0.156 | 0.723 | 0.921 | 0.340 | 0.462 | 0.811 | 0.218 | 0.539 | 0.450 | 0.929 | 0.559 | 0.363 | 0.811 | 0.482 | 0.302 | 0.854 |
| *WNK1* | SNP43_rs2255390 | 0.879 | 0.389 | 0.232 | 0.197 | 0.417 | 0.186 | 0.298 | 0.911 | 0.097 | 0.272 | 0.411 | 0.325 | 0.850 | 0.875 | 0.622 | 0.618 | 0.913 | 0.463 |
| *NR2F2-AS1* | SNP45_rs2398162 | 0.607 | 0.528 | 0.899 | 0.702 | 0.476 | 0.806 | 0.982 | 0.838 | 0.741 | 0.107 | 0.303 | 0.085 | 0.381 | 0.647 | 0.281 | 0.207 | 0.473 | 0.140 |
| *AGTR1* | SNP46_rs2638360 | 0.594 | 0.358 | 0.327 | 0.598 | 0.780 | **0.007** | 0.502 | 0.942 | **0.010** | 0.773 | 0.806 | 0.801 | 0.479 | 0.587 | 0.426 | 0.479 | 0.582 | 0.440 |
| *NEDD4L* | SNP50_rs4149601 | 0.118 | 0.184 | 0.182 | 0.703 | 0.866 | 0.422 | 0.094 | 0.162 | 0.133 | 0.821 | 0.861 | 0.805 | 0.539 | 0.481 | 0.972 | 0.631 | 0.564 | 0.917 |
| *CYBA* | SNP51_rs4673 | **0.180** | **0.019** | 0.171 | 0.554 | 0.834 | 0.289 | **0.005** | **0.033** | **0.006** | **1.60E-05^b^** | **2.80E-05 ^b^** | **0.016** | 0.994 | 0.771 | 0.571 | 0.195 | 0.312 | 0.224 |
| *NR3C2* | SNP91_rs7694064 | 0.933 | 0.871 | 0.909 | 0.475 | 0.195 | 0.421 | 0.409 | 0.229 | 0.725 | 0.942 | 0.799 | 0.497 | 0.709 | 0.566 | 0.827 | 0.725 | 0.667 | 0.999 |
| *NR3C2* | SNP92_rs6856803 | 0.621 | 0.962 | 0.391 | 0.743 | 0.659 | 0.924 | 0.315 | 0.468 | 0.358 | 0.061 | 0.093 | 0.167 | 0.133 | 0.566 | 0.060 | 0.449 | 0.998 | 0.213 |
| *SELE* | SNP93_rs4656704 | 0.454 | 0.363 | 0.827 | **0.030** | **0.023** | **0.264** | 0.118 | 0.057 | 0.652 | 0.144 | 0.189 | 0.297 | 0.742 | 0.956 | 0.580 | 0.878 | 0.727 | 0.863 |
| *SELE* | SNP94_rs6427212 | 0.654 | 0.767 | 0.622 | 0.101 | 0.116 | 0.338 | 0.339 | 0.121 | 0.586 | **0.052** | **0.044** | **0.384** | 0.774 | 0.902 | 0.391 | 0.718 | 0.463 | 0.643 |
| *SELE* | SNP95_rs5368 | 0.923 | 0.846 | 0.888 | 0.393 | 0.344 | 0.810 | 0.437 | 0.183 | 0.491 | **0.017** | **0.011** | **0.397** | 0.612 | 0.904 | 0.146 | 0.777 | 0.374 | 0.306 |
| *SCNN1A* | SNP96_rs11064153 | 0.372 | 0.408 | 0.559 | 0.528 | 0.831 | 0.319 | 0.858 | 0.710 | 0.836 | 0.109 | 0.125 | 0.332 | 0.428 | 0.413 | 0.700 | 0.830 | 0.797 | 0.962 |
| *SCNN1G* | SNP97_rs4401050 | 0.436 | 0.459 | 0.686 | 0.373 | 0.517 | 0.209 | 0.187 | 0.258 | 0.236 | 0.792 | 0.882 | 0.083 | 0.822 | 0.656 | 0.419 | 0.906 | 0.661 | 0.215 |
| *CACNA1C* | SNP98_rs8182538 | 0.453 | 0.784 | 0.107 | 0.597 | 0.708 | 0.619 | 0.820 | 0.831 | 0.524 | 0.216 | 0.085 | 0.829 | 0.643 | 0.816 | 0.285 | 0.972 | 0.467 | 0.379 |

^a^ΔSBP1, ΔDBP1, and ΔMAP1 were defined as the BP after acute salt loading for 2 hours minus BP at baseline; ΔSBP2, ΔDBP2, and ΔMAP2 were defined as the BP after taking oral furosemide for 2 hours minus BP before taking oral furosemide.

^b^FDR *P* value< 0.05;*P* value < 0.05 are bolded

**Table S4**. Multiple logistic regression analysis of associations between 11 SNPs and SS

| Gene /dpSNP | Model | Genotype | SR^a^ n (%) | SS^a^ n (%) | *p* ^b^ | OR (95% CI) ^b^ |
| --- | --- | --- | --- | --- | --- | --- |
| PRKG1 | Additive | GG | 170(11.7) | 74(13.1) | 0.261 | 1.197 (0.875-1.398) |
| rs1904694 |  | GA | 651(44.2) | 267(47.1) | 0.263 | 1.134 (0.921-1.398) |
|  |  | AA | 627(43.3) | 226(39.9) | - | 1 |
|  | Dominant | GG+GA *vs.* AA | 821(56.7)/627(43.3) | 341(60.1)/226(39.9) | 0.173 | 1.147 (0.941-1.398) |
|  | Recessive | GG *vs.* GA+AA | 170(11.1)/1278(88.3) | 74(13.1)/493(86.9) | 0.448 | 1.120 (0.836-1.500) |
| PRKG1 | Additive | AA | 292(20.3) | 128(22.5) | **0.043** | 1.337 (1.010-1.771) |
| rs7897633 |  | AC | 706(49.2) | 298(52.4) | **0.031** | 1.292 (1.024-1.630) |
|  |  | CC | 437(30.5) | 143(25.1) | - | 1 |
|  | Dominant | AA+AC *v*s. CC | 998(69.5)/437(30.5) | 426(74.9)/143(25.1) | **0.018** | 1.305 (1.047-1.627) |
|  | Recessive | AA *vs*. AC+CC | 292(20.3)/1143(79.7) | 128(22.5)/441(77.5) | 0.298 | 1.133 (0.895-1.434) |
| CYBA | Additive | AA | 289(20.35) | 126(22.3) | **0.006** | 2.410 (1.288-4.510) |
| rs4673 |  | AG | 695(48.94) | 295(52.5) | 0.080 | 1.758 (0.935-3.304) |
|  |  | GG | 436(30.71) | 142(25.2) | - | 1 |
|  | Dominant | AA+AG *vs.* GG | 984(69.30)/436(30.70) | 421(74.78)/142(25.22) | 0.307 | 1.108 (0.910-1.348) |
|  | Recessive | AA *vs.* AG+GG | 289(20.35)/1131(79.65) | 126(22.38)/437(77.62) | **0.006** | 1.673 (1.155-2.421) |
| SLC8A1 | Additive | AA | 135(9.3) | 45(7.9) | 0.278 | 1.225 (0.849-1.765) |
| rs11893826 |  | AG | 637(44.0) | 249(43.8) | 0.392 | 1.175 (0.813-1.698) |
|  |  | GG | 675(46.6) | 275(48.3) | - | 1 |
|  | Dominant | AA+AG *vs.* GG | 772(53.4)/675(46.6) | 294(51.7)/275(48.3) | 0.493 | 0.934 (0.769-1.135) |
|  | Recessive | AA *vs.* AG+GG | 135(9.3)/1312(90.7) | 45(7.9)/524(92.1) | 0.310 | 0.833 (0.586-1.185) |
| AGTR1 | Additive | AA | 833(58.66) | 315(55.95) | 0.041 | 2.165 (1.032-4.545) |
| rs2638360 |  | GA | 510(35.92) | 198(35.17) | 0.769 | 1.042 (0.792-1.371) |
|  |  | GG | 77(5.42) | 50(8.88) | - | 1 |
|  | Dominant | GG+GA vs. AA | 587(41.34)/833(58.66) | 248(44.05)/315(55.95) | 0.402 | 1.118 (0.861-1.452) |
|  | Recessive | GG *vs.* GA+AA | 77(5.42)/1343(94.58) | 50(8.88)/513(91.12) | 0.043 | 2.150 (1.026-4.506) |
| SELE | Additive | GG | 235(16.3) | 79(13.8) | 0.160 | 0.806 (0.597-1.088) |
| rs4656704 |  | GA | 685(47.4) | 277(48.3) | 0.732 | 0.964 (0.780-1.190) |
|  |  | AA | 525(36.3) | 218(38.0) | - | 1 |
|  | Dominant | GG+GA *vs* AA | 920(63.7)/525(36.6) | 356(62.0)/218(38.0) | 0.436 | 0.924 (0.756-1.128) |
|  | Recessive | GG *vs*. GA+AA | 235(16.3)/1210(83.7) | 79(13.8)/495(86.2) | 0.167 | 0.823 (0.625-1.085) |
| SELE | Additive | AA | 135(9.4) | 48(8.4) | 0.379 | 0.851(0.595-1.218) |
| rs6427212 |  | AG | 646(44.8) | 248(43.3) | 0.413 | 0.919(0.750-1.126) |
|  |  | GG | 662(45.9) | 275(48.2) | - | 1 |
|  | Dominant | AA+AG *vs* GG | 781(54.1)/662(45.9) | 296(51.8)/275(48.2) | 0.324 | 0.907(0.747-1.101) |
|  | Recessive | AA *vs*. AG+GG | 135(9.4)/1308(90.6) | 48(8.4)/523(91.6) | 0.496 | 0.887(0.628-1.253) |
| SELE | Additive | AA | 110(7.6) | 37(6.5) | 0.345 | 0.980(0.801-1.199) |
| rs5368 |  | AG | 594(41.3) | 237(41.4) | 0.843 | 0.980(0.801-1.199) |
|  |  | GG | 736(51.1) | 299(52.2) | - | 1 |
|  | Dominant | AA+AG *vs* GG | 736(51.1)/704(48.9) | 299(52.2)/274(47.8) | 0.648 | 1.046(0.862-1.270) |
|  | Recessive | AA *vs*. AG+GG | 110(7.6)/1330(92.4) | 37(6.5)/536(93.5) | 0.356 | 1.199(0.815-1.765) |
| CYP4A11 | Additive | GG | 70(4.9) | 23(4.1) | **0.018** | 1.285(1.044-1.581) |
| rs1126742 |  | GA | 456(31.8) | 210(37.3) | 0.765 | 0.928(0.569-1.514) |
|  |  | AA | 906(63.3) | 330(58.6) | - | 1 |
|  | Dominant | GG+GA *vs* A | 526(36.7)/906(63.3) | 233(41.4)/330(58.6) | **0.037** | 1.238(1.013-1.512) |
|  | Recessive | GG *vs*. GA+AA | 70(4.9)/1362(95.1) | 23(4.1)/540(95.9) | 0.502 | 0.847(0.523-1.374) |
| BCAT1 | Additive | AA | 22(1.6) | 10(1.7) | 0.733 | 1.043(0.819-1.329) |
| rs7961152 |  | AC | 299(21.7) | 120(22.3) | 0.627 | 1.207(0.565-2.575) |
|  |  | CC | 1059(76.7) | 408(75.8) |  |  |
|  | Dominant | AA+AC *vs* CC | 321(23.3)/1059(76.7) | 130(24.2)/408(75.8) | 0.660 | 1.054(0.833-1.333) |
|  | Recessive | AA *vs*. AC+CC | 22(1.6)/1358(98.4) | 10(1.9)/528(98.1) | 0.644 | 1.195(0.561-2.546) |
| VSNL1 | Additive | GG | 28(2.0) | 14(2.5) | 0.385 | 0.741(0.377-1.456) |
| rs16983422 |  | GA | 327(22.9) | 126(22.1) | 0.439 | 0.773(0.402-1.484) |
|  |  | AA | 1075(75.2) | 429(75.4) |  |  |
|  | Dominant | GG+GA *vs* AA | 355(24.8)/1075(75.2) | 140(24.6)/429(75.4) | 0.896 | 0.985(0.786-1.235) |
|  | Recessive | GG *vs*. GA+AA | 28(2.0)/1402(98.0) | 14(2.5)/555(97.5) | 0.421 | 1.307(0.682-2.505) |

Abbreviations: SR, salt-resistance; SS, salt-sensitivity; FDR, false discovery rate. ^a^Numbers are frequencies and percentage. ^b^ Adjusted for age, gender, TG, FBG, WHR, salt-eating habit, daily walking times and hypertension.

*P* value < 0.05 are bolded; *FDR-*P*< 0.05

**Table S5. The effects of CYBA/rs4673 and AGTR1/rs2638360 interaction on the risk of SS**

| **SNPs** | **CYBA/rs4673** | | | | | | | | | **Interaction analysis** |
| --- | --- | --- | --- | --- | --- | --- | --- | --- | --- | --- |
|  | **GG** | | | **GA** | | | **AA** | | |  |
| **AGTR1/rs2638360** | **SS/** | **OR** | ***P*** | **SS/** | **OR** | ***P*** | **SS/** | **OR** | ***P*** | ***P*_multiplicative_** |
|  | **SR** | **(95%CI)** |  | **SR** | **(95%CI)** |  | **SR** | **(95%CI)** |  |  |
| AA | 72/241 | 1.000 | - | 174/415 | 1.396 | **0.040** | 69/177 | 1.280 | 0.210 | 0.004 |
|  |  |  |  |  | (1.015, 1.920) |  |  | (0.870, 1.885) |  |  |
| GA | 52/167 | 1.030 | 0.887 | 100/244 | 1.363 | 0.086 | 46/99 | 1.547 | 0.052 |  |
|  |  | (0.684, 1.552) |  |  | (0.957, 1.941) |  |  | (0.996, 2.403) |  |  |
| GG | 18/28 | 1.976 | **0.044** | 21/36 | 1.98 | **0.026** | 11/13 | 2.819 | **0.017** |  |
|  |  | (1.019, 3.832) |  |  | (1.085, 3.612) |  |  | (1.204, 6.602) |  |  |

All *P* values were calculated by logistic regression model after adjusting for gender and ageexcept the multiplicative interaction analysis which was calculated using the bootstrapping test of goodness-of-fit.

**Table S6. Phenotypes associated with SSBP-related SNPs using GeneAtlas**

| **Gene** | **SNP** | **Associated phenotypes ordered by *P* value** | **Strongest phenotype** | ***P* value for Strongest phenotype** |
| --- | --- | --- | --- | --- |
| CYBA | SNP51_rs4673 | Standing height; Mean corpuscular volume; Trunk fat-free mass; Sitting height; Whole body fat-free mass; Basal metabolic rate | Standing height | **1.69E-10** |
| SLC8A1 | SNP6_rs11893826 | Essential hypertension; hypertension | Essential hypertension | **1.30E-04** |
| SELE | SNP93_rs4656704 | Monocyte count; Monocyte percentage; Ischaemic heart diseases; Waist circumference / Hip circumference | Monocyte count | **2.97E-10** |
| SELE | SNP95_rs5368 | Monocyte count | Monocyte count | **3.97E-09** |

**Table S7. *P* value (before/after adjusted) for association between** **SSBP phenotypes and risk factors**

| **Risk  facotrs** | ***P* values (before/after adjusted^a^)** | | | | | |
| --- | --- | --- | --- | --- | --- | --- |
|  | **ΔSBP1^b^** | **ΔDBP1 ^b^** | **ΔMAP1 ^b^** | **ΔSBP2 ^b^** | **ΔDBP2 ^b^** | **ΔMAP2 ^b^** |
| WHR | 0.821/0.584 | 0.838/0.886 | 0.925/0.756 | 0.057/**0.048** | 0.388/0.409 | 0.179/0.147 |
| TG | 0.547/0.859 | 0.097/0.296 | 0.687/0.823 | 0.525/0.122 | **0.049**/0.112 | 0.053/0.062 |
| TC | 0.818/0.712 | 0.539/0.506 | 0.906/0.659 | 0.069/0.187 | 0.219/0.657 | 0.598/0.389 |
| HDL-C | **0.002/0.004*** | 0.275/0.525 | 0.075/0.348 | **2.22E-04/1.69E-04*** | 0.232/0.978 | **0.029/0.011** |
| LDL-C | **0.009/0.003*** | 0.365/0.246 | 0.078/0.722 | **0.020/0.013** | 0.315/0.602 | 0.827/0.182 |
| FBG | **0.033/0.043** | **0.025/0.020** | **0.017/0.003*** | 0.510/0.294 | 0.770/0.475 | 0.646/0.863 |

^a^Adjusted for age, gender, TG, FBG, WHR, salt-eating habit, daily walking times, and hypertension.

^b^ΔSBP1, ΔDBP1, and ΔMAP1 were defined as the BP after acute salt loading for 2 hours minus BP at baseline; ΔSBP2, ΔDBP2, and ΔMAP2 were defined as the BP after taking oral furosemide for 2 hours minus BP before taking oral furosemide.

*P* value < 0.05 are bolded.

^*^The FDR was used for multiple testing, FDR*P*≤ 0.05 were considered statisticallysignificant.

**Table S8. *P* value for association between SNPs and SSBP risk factors**

| **SNP** | **Genetic**  **Model** | **WHR** | **TC** | **TG** | **HDL-C** | **LDL-C** | **FBG** |
| --- | --- | --- | --- | --- | --- | --- | --- |
|  |  | ***P*** | ***P*** | ***P*** | ***P*** | ***P*** | ***P*** |
| BCAT1/rs7961152 | additive | 0.168 | 0.200 | 0.452 | 0.354 | 0.679 | 0.595 |
|  | dominant | 0.159 | 0.234 | 0.432 | 0.378 | 0.729 | 0.574 |
|  | recessive | 0.461 | 0.315 | 0.863 | 0.680 | 0;803 | 0.996 |
| PRKG1/rs7897633 | additive | 0.393 | 0.103 | **0.030** | 0.283 | 0.296 | **0.001*** |
|  | dominant | 0.693 | 0.222 | **0.028** | 0.234 | 0.297 | **0.001*** |
|  | recessive | 0.097 | 0.276 | 0.127 | 0.489 | 0.438 | **0.034** |
| SLC8A1/rs11893826 | additive | 0.330 | 0.476 | 0.946 | 0.244 | 0.757 | 0.761 |
|  | dominant | 0.141 | 0.365 | 0.702 | 0.276 | 0.995 | 0.983 |
|  | recessive | 0.736 | 0.964 | 0.579 | 0.494 | 0.498 | 0.464 |
| AGTR1/rs2638360 | additive | 0.805 | 0.055 | 0.177 | 0.755 | 0.494 | 0.713 |
|  | dominant | 0.768 | 0.062 | 0.636 | 0.893 | 0.279 | 0.684 |
|  | recessive | 0.788 | 0.413 | **0.001*** | 0.622 | 0.477 | 0.894 |
| CYBA/rs4673 | additive | 0.514 | **2.269E-21*** | 0.817 | **2.211E-104*** | **1.610E-64*** | 0.351 |
|  | dominant | 0.312 | **2.5796E-21*** | 0.550 | **4.8731E-115*** | **9.6054E-69*** | 0.419 |
|  | recessive | 0.926 | **1.3595E-8*** | 0.991 | **7.6347E-17*** | **1.2296E-13*** | 0.672 |
| SELE/rs6427212 | additive | 0.787 | 0.237 | 0.645 | 0.583 | 0.548 | 0.471 |
|  | dominant | 0.634 | 0.346 | 0.387 | 0.945 | 0.973 | 0.975 |
|  | recessive | 0.648 | 0.518 | 0.715 | 0.271 | 0.108 | 0.076 |
| SELE/rs5368 | additive | 0.982 | 0.698 | 0.920 | 0.913 | 0.565 | 0.304 |
|  | dominant | 0.823 | 0.590 | 0.925 | 0.963 | 0.831 | 0.875 |
|  | recessive | 0.656 | 0.607 | 0.809 | 0.796 | 0.304 | **0.025** |
| VSNL1/rs16983422 | additive | 0.382 | 0.629 | 0.206 | 0.768 | 0.867 | 0.994 |
|  | dominant | 0.413 | 0.752 | 0.574 | 0.903 | 0.754 | 0.778 |
|  | recessive | 0.594 | 0.720 | **0.014** | 0.194 | 0.632 | 0.373 |
| SELE/rs4656704 | additive | 0.520 | 0.191 | 0.639 | 0.949 | 0.846 | 0.163 |
|  | dominant | 0.492 | 0.487 | 0.528 | 0.827 | 0.921 | 0.318 |
|  | recessive | 0.925 | 0.175 | 0.930 | 0.662 | 0.452 | 0.155 |
| PRKG1/rs1904694 | additive | 0.353 | 0.106 | 0.114 | 0.246 | 0.341 | 0.066 |
|  | dominant | 0.214 | 0.367 | 0.175 | 0.361 | 0.437 | 0.222 |
|  | recessive | 0.835 | 0.147 | 0.168 | 0.284 | 0.589 | **0.038** |
| CYP4A11/rs1126742 | additive | 0.162 | **0.021** | **0.038** | 0.259 | 0.223 | 0.692 |
|  | dominant | 0.234 | 0.085 | **0.045** | 0.522 | 0.516 | 0.103 |
|  | recessive | 0.935 | 0.045 | 0.343 | 0.085 | 0.076 | 0.127 |

*P* value < 0.05 are bolded.

*The FDRwas used for multiple testing, FDR*P*≤ 0.05 were considered statistically significant.


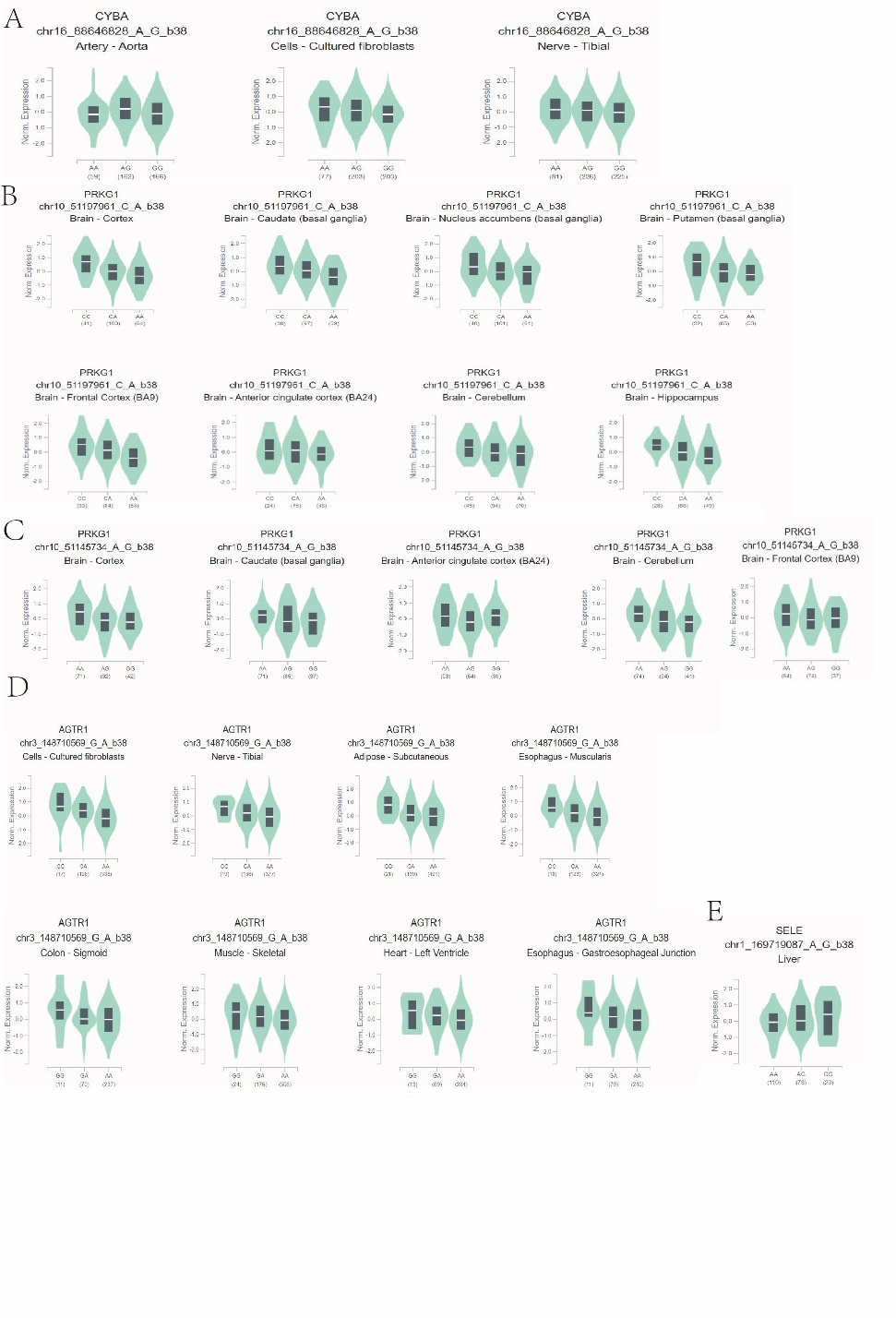


**Figure S1**. The Violin plot shows thecorrelation between SNP and gene expression in the GTEx database. (A) Expression of CYBA stratified by rs4673genotypes; (B)Expression of PRKG1 stratified by rs7897633 genotypes; (C) Expression of PRKG1stratified by rs1904694 genotypes; (D) Expression of AGTR1 stratified by rs2638360genotypes; (E) Expression of SELE stratified byrs4656704

genotypes.


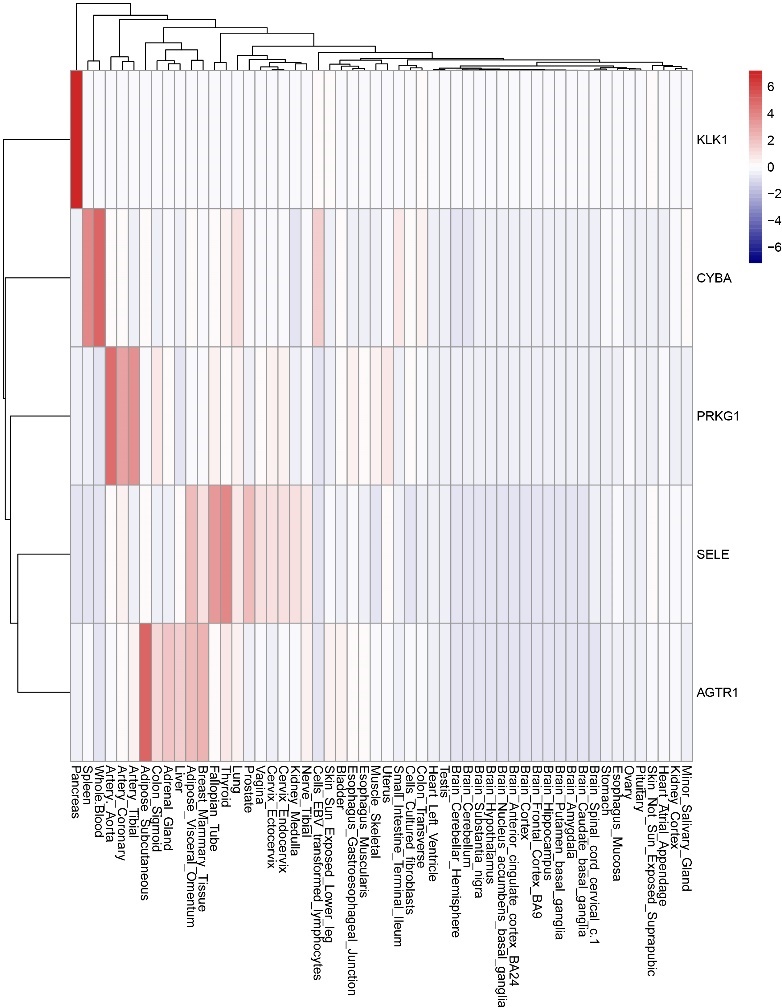


**Figure S2.**Standardized median gene expressions across 54 tissues for genes mapped to SSBP-related SNP.

Blue spectrum color shows low expression, white spectrum represents moderate expression and red to yellow spectrum show high expression
